# Dual peptides-modified cationic liposomes for enhanced Lung cancer gene therapy by a gap junction regulating strategy

**DOI:** 10.1186/s12951-023-02242-1

**Published:** 2023-12-09

**Authors:** Ziyu Zhao, Wenhao Wang, Guanlin Wang, Zhengwei Huang, Liping Zhou, Li Lin, Yueling Ou, Wanzhen Huang, Xuejuan Zhang, Chuanbin Wu, Liang Tao, Qin Wang

**Affiliations:** 1 Department of Pharmacology, Zhongshan School of Medicine, Sun Yat-Sen University, Guangzhou, 510080 PR China; 2 State Key Laboratory of Bioactive Molecules and Druggability Assessment, Jinan University, Guangzhou, Guangdong 510632 PR China; 3 College of Pharmacy, Jinan University, Guangzhou, Guangdong 510632 PR China; 4 School of Pharmaceutical Sciences, Sun Yat-Sen University, Guangzhou, Guangdong 510006 PR China; 5 Nanchang Research Institute, Sun Yat-Sen University, Nanchang, Jiangxi 330096 PR China

**Keywords:** Lung cancer, Gene therapy, Gap junction regulation, Dry powder inhaler, miR-34a

## Abstract

**Background:**

Gene therapy for lung cancer has emerged as a novel tumor-combating strategy for its superior tumor specificity, low systematical toxicity and huge clinical translation potential. Especially, the applications of microRNA shed led on effective tumor ablation by directly interfering with the crucial gene expression, making it one of the most promising gene therapy agents. However, for lung cancer therapy, the microRNA treatment confronted three bottlenecks, the poor tumor tissue penetration effect, the insufficient lung drug accumulation and unsatisfied gene transfection efficiency. To address these issues, an inhalable RGD-TAT dual peptides-modified cationic liposomes loaded with microRNA miR-34a and gap junction (GJ) regulation agent all-trans retinoic acid (ATRA) was proposed, which was further engineered into dry powder inhalers (DPIs).

**Results:**

Equipped with a rough particle surface and appropriate aerodynamic size, the proposed RGD-TAT-CLPs/ARTA@miR-34a DPIs were expected to deposit into the deep lung and reach lung tumor lesions guided by targeting peptide RGD. Assisted by cellular transmembrane peptides TAT, the RGD-TAT-CLPs/ARTA@miR-34a was proven to be effectively internalized by cancer cells, enhancing gene transfection efficiency. Then, the GJ between tumor cells was upregulated by ARTA, facilitating the intercellular transport of miR-34a and boosting the gene expression in the deep tumor.

**Conclusion:**

Overall, the proposed RGD-TAT-CLPs/ARTA@miR-34a DPIs could enhance tumor tissue penetration, elevate lung drug accumulation and boost gene transfection efficiency, breaking the three bottlenecks to enhancing tumor elimination in vitro and in vivo. We believe that the proposed RGD-TAT-CLPs/ARTA@miR-34a DPIs could serve as a promising pulmonary gene delivery platform for multiple lung local disease treatments.

**Supplementary Information:**

The online version contains supplementary material available at 10.1186/s12951-023-02242-1.

## Introduction

Lung cancers have become one of the most severe challenges for human beings with about 2.2 million new cases and 1.8 million deaths in 2020 [1]. Among these cases, non-small cell lung cancer (NSCLC) constitutes around 85% of all lung cancer patients, presenting a critical clinical burden [2]. Until now, the first-line clinical treatment for early NSCLC is surgery, while most patients in the advanced stage are not suitable for surgical excision [3]. Consequently, traditional chemotherapy has become the predominant choice for this patient population [4]. For decades of clinical practice, serval nonnegligible issues have been revealed in chemotherapy, including systematic side effects, high drug resistance, difficulty in preventing tumor metastasis and unsatisfied survival rate. Therefore, there exists an urgent necessity to explore novel approaches for treating NSCLC [5].

Recently, several new lung tumor elimination approaches have been proposed with improved treatment efficiency, such as immunotherapy [6], ferroptosis therapy [7] and gene therapy [8]. Therein, burgeoning efforts on gene therapy have been made due to its high tumor specificity, low systematical toxicity and desired clinical translation potential [9]. It has been recorded that about 2000 gene therapy agents have reached the clinical trial, and a few of them have already been on the market, such as Gendicine®, Oncorine® and Imlygic® [9–11]. Hence, the boosting development of gene therapy provides widespread opportunities against NSCLC.

Among all kinds of gene therapy agents, microRNA (miRNA), a noncoding single-stranded RNA molecule of approximately 21–25 nucleotides in length encoded by an endogenous gene, has attracted worldwide attention [12]. By interfering with the crucial gene expression in tumorigenesis, the employment of miRNA is expected to directly inhibit tumor growth and break the limits of traditional chemotherapy [13, 14]. Currently, there is a significant surge in interest surrounding miR-34a (miRNA-34a), which plays a pivotal role in key molecular signaling mechanisms across various tumor models, including NSCLC [15]. Studies have reported that introducing miR-34a mimics into the NSCLC model triggers upregulation of p53 and downregulation of the B-cell lymphoma-2 (Bcl-2) gene, thereby enhancing tumor apoptosis and facilitating effective tumor ablation [16, 17]. Thus, the employment of miR-34a is promising in the treatment of NSCLC.

Nevertheless, the miR-34a application in NSCLC still is limited by three key bottlenecks, (1) the poor tumor tissue penetration effect, (2) the insufficient lung drug accumulation and (3) unsatisfied gene transfection efficiency. The breakthrough of these three bottlenecks is expected to pronouncedly boost the real-world clinical application of miR-34a.

For bottleneck (1): Unlike normal organ tissues, the tumor tissues are equipped with intense intertumoral barriers due to their abnormal blood vessels, dense extracellular matrix (ECM) and elevated interstitial fluid pressure (IFP), which severely hamper the deep tumor transportation of miR-34a, resulting in undesired therapeutical efficiency [18]. To solve this issue, the endogenous intercellular communication mechanisms of tumor cells are regarded as potential targets to improve miR-34a distribution in deep tumor. Gap junctions (GJs), widely recognized as intercellular transport channels, have been hypothesized to facilitate gene agent penetration [19, 20]. GJs consist of two connexons composed of connexin (Cx) proteins and play a crucial role in facilitating the transport of ions, second messengers, small molecule nutrients, and metabolites between neighboring cells [21]. This intercellular communication mechanism is essential for the regulation of tissue homeostasis, growth and proliferation [22, 23]. Recent studies have revealed the damaged GJ functions in NSCLC, which is marked by the downregulation of Cx43, a critical GJ-composing protein [24]. On one hand, the impaired function of gap junctions (GJ) has been implicated in promoting abnormal tumor development and metastasis by inhibiting the monitoring and regulation between tumor cells and normal cells. On the other hand, the limited GJ function may also impede drug delivery from the superficial layer to the deep layer of tumor cells. The existence of a phenomenon known as “by-stander effect” enables transportation of drugs themselves and drug-triggered cell death signals through GJ among tumor cells, ultimately inducing an expansion of cell death wave. However, it has been observed that this process is severely inhibited in NSCLC tissue [24]. Hence, the tumor GJ reconstruction and recovery strategy were considered a promising way to break the intertumoral delivery barrier, further boosting the treatment efficiency of gene therapy. In this work, the classical and effective GJ regulation agent all-trans retinoic acid (ATRA) was chosen, which could enhance the GJ function by upregulating the Cx43 expression [25]. Thus, the co-delivery of ATRA and miR-34a was expected to exert a satisfactory poor tumor tissue penetration effect for miR-34a.

For bottleneck (2): An ideal NSCLC gene therapy scenario should not only involve orchestrated strategies for GJ function regulation but also be equipped with a lung-specific drug delivery system. The current conventional approaches to drug administration, such as oral intake and intravenous injection, fail to meet the specific requirements for targeted drug accumulation in the lungs. Therefore, the introduction of an innovative pulmonary drug delivery system holds great promise for enhancing treatment strategies for non-small cell lung cancer (NSCLC), particularly in terms of gene therapy targeting patterns and mitigating low enzyme activity associated with this system. [26]. By virtue of sufficient lung lesion drug accumulation and reduced systematical distribution, noninvasive pulmonary delivery has been successfully developed in the treatment of asthma, cystic fibrosis, pneumonia, chronic obstructive pulmonary diseases and other lung diseases [27–29]. As a potent pulmonary delivery approach, dry powder inhalers (DPIs) can deliver micro-sized particles towards lung lesions, where the drugs were loaded in solid-state particles to maintain a high drug loading and stability [30]. DPIs have been successfully introduced into the market for the treatment of lung diseases, highlighting their significant potential for industrialization. Consequently, the development of co-delivered DPIs incorporating miR-34a and ARTA holds substantial promise in advancing lung gene therapy.

For bottleneck (3): To effectively transfect miR-34a to tumor cells, the appropriate on-demand gene vectors with tumor-targeting capacity are also essential. Viral vectors and non-viral vectors represent the mainstream choices for gene delivery. Among them, the non-viral ones were considered to possess higher transfection efficiency and safety [31, 32]. As one of the most widely employed non-viral vectors, the cationic liposomes (CLPs) vectors have been registered in many clinical trials for NSCLC, and CLPs-based coronavirus disease 2019 (COVID-19) mRNA vaccines have also been employed worldwide in the current pandemic, suggesting their ample application potential [33–35]. The negatively-charged miRNA can be easily adsorbed onto the positively-charged CLPs to form a stable liposome-gene complex, which can subsequently be delivered intracellularly without undergoing enzyme degradation. Moreover, the biocompatible nature of CLPs carriers in the respiratory tract further facilitates their application in pulmonary delivery. Therefore, the preparation of CLPs carriers loaded with miR-34a and ARTA holds great promise for enhancing NSCLC gene therapy through GJ regulation strategy. However, owing to the strong electrostatic interaction between CLPs and mammal cell membranes, CLPs is hard to differentiate cancer cells from normal cells, which may induce unexpected side effects of cargoes in pulmonary delivery applications [36]. Worse still, merely depending on the cationic nature of CLPs to promote tumor cell uptake is not effective enough, which may cause severe off-target issues. Thus, a robust tumor cell targeting and uptake-improving strategy is critical to enhancing tumor cell miRNA transfection efficiency. Here, the tumor cell membrane αvβ3 targeting peptide arginyl-glycyl-aspartic acid (RGD) combined with cellular transmembrane peptides (CPPs) TAT (YGRKKRRQRRR) was introduced to break this bottleneck [37, 38]. The RGD peptide could serve as a “guide molecule” to recognize tumor cells while the TAT could assist the tumor uptake of CLPs. This dual peptide-modified CLPs was expected to achieve ultrahigh tumor targeting and internalization effects, boosting the GJ function regulating strategy enhanced gene therapy and reducing the side effects.

**Scheme 1:**
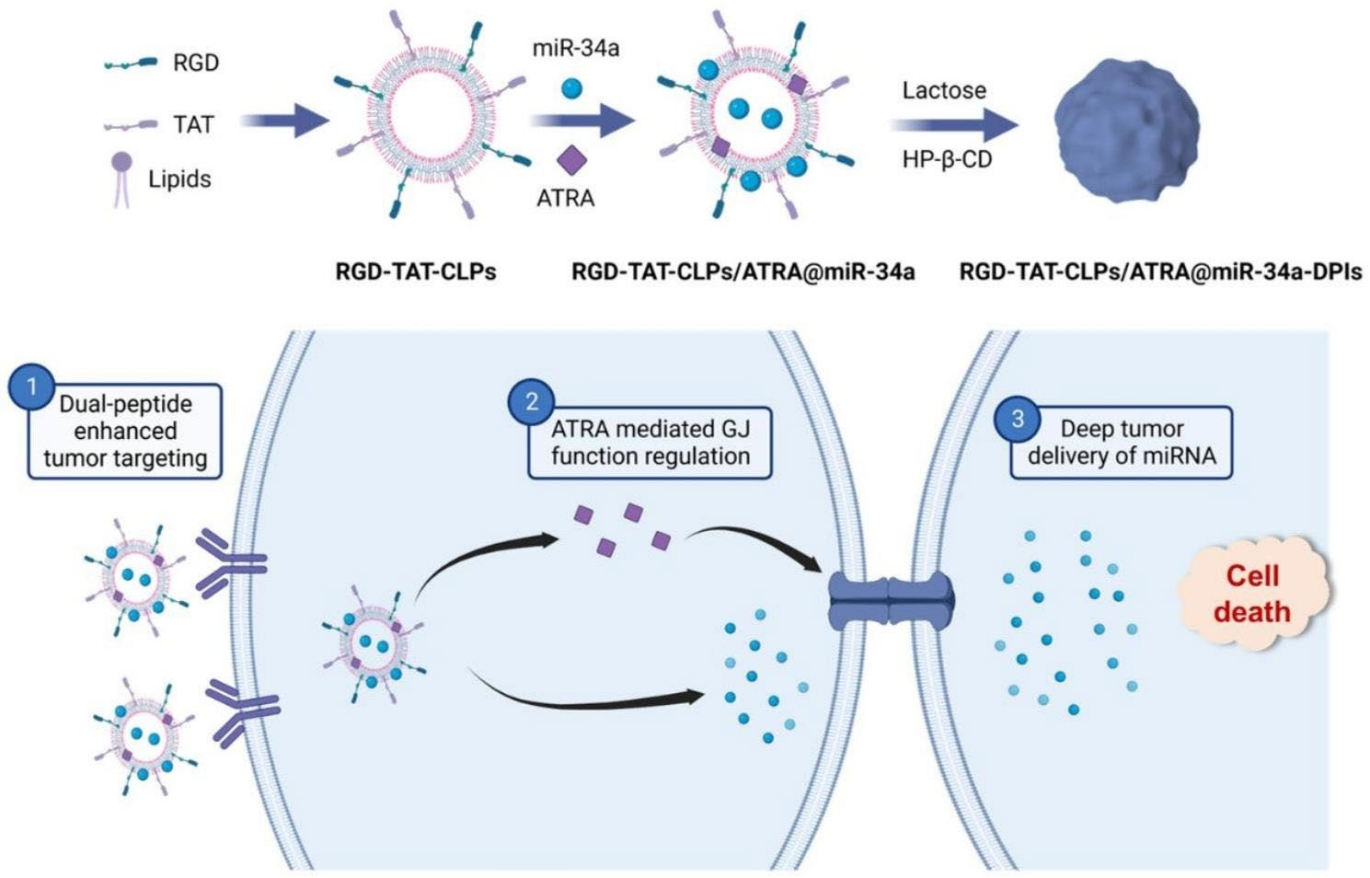
The schematical illustration of the inhalable dual peptides-modified cationic liposomes for enhanced lung cancer gene therapy by a gap junction regulating strategy. ? The enhanced tumor targeting of RGD-TAT-CLPs/ARTA@miR-34a mediated by dual peptide modification. ? The released ARTA would upregulate the expression of GJ and promote the intercellular transport of miR-34a. ? The miR-34a would be transported through GJ and achieve deep tumor delivery, inducing effective tumor death

In this work, an RGD-TAT dual peptide modified miR-34a and ARTA co-loaded CLPs (RGD-TAT-CLPs/ARTA@miR-34a) was proposed for the GJ regulating strategy enhanced gene therapy of NSCLC, which was then engineered into DPIs (Scheme 1). Upon pulmonary delivery, the RGD-TAT-CLPs/ARTA@miR-34a DPIs with fine aerosolization performance could deposit into deep lung lesions and release RGD-TAT-CLPs/ARTA@miR-34a. Guided by RGD peptide, the released RGD-TAT-CLPs/ARTA@miR-34a could accurately recognize tumor cells and be efficiently internalized with the assistance of TAT. After being intracellular delivered, the ARTA could upregulate the expression of Cx43 to regulate GJ function while the miR-34a could downregulate the Bcl-2 to induce cell apoptosis. Due to the enhanced GJ function, the miR-34a could effectively transport between tumor cells and distribute into deep tumor tissue, finally boosting the gene therapy in NSCLC. The proposed drug delivery system has been demonstrated to effectively facilitate NSCLC gene therapy through enhanced miR-34a deep tumor transportation, increased accumulation of drugs in tumor lesions, and improved efficiency of gene transfection. We firmly believe that the RGD-TAT-CLPs/ARTA@miR-34a DPIs proposed herein could serve as a robust paradigm for NSCLC gene therapy.

## Results and discussion

### Preparation and characterization of RGD-TAT-CLPs/ATRA@miR-34a

**Fig. 1:**
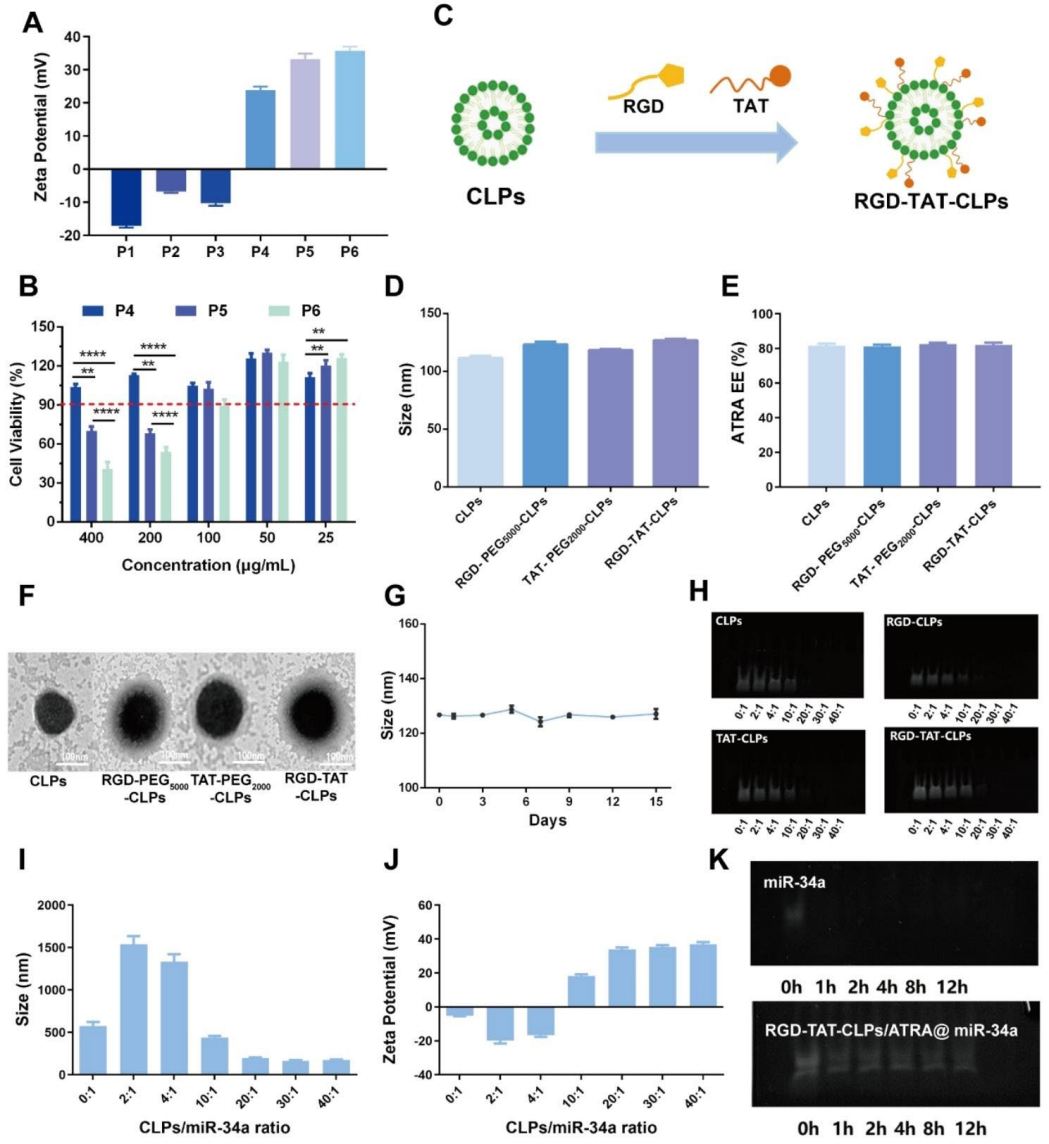
Preparation and characterization of RGD-TAT-CLPs/ATRA@miR-34a: (**A-B**) Zeta Potential (**A**) and cytotoxicity on Base-2b cells (**B**) of different CLPs formulations. (**C**) The schematical illustration of RGD-TAT-CLPs preparation. (**D-F**) The particle size (**D**), ARTA EE (**E**) and TEM morphology (**F**) of CLPs, RGD-PEG_5000_-CLPs, TAT-PEG_2000_-CLPs and RGD-TAT-CLPs. (**G**) The stability of RGD-TAT-CLPs. (**H**) The gel electrophoresis images of free miR-34a in the preparation of CLPs@miR-34a with different targeting peptides and different miR-34a loading. (**I-J**) The particle size (**I**) and Zeta Potential (**J**) of RGD-TAT-CLPs/ATRA@miR-34a with different miR-34a loading. (**K**) The gel electrophoresis images of miR-34a and RGD-TAT-CLPs/ATRA@miR-34a under FBS condition within 12 h. (*n* = 3)

The proposed RGD-TAT-CLPs/ATRA@miR-34a was prepared by a thin layer dispersion method. Firstly, a series of parameters were optimized to construct the blank liposomes with superior particle size and encapsulation efficiency (EE), including egg phospholipids (EP)/cholesterol (Chol) ratio, hydration volume, ultrasonic power and ultrasonic time (Figs. S1-S4). Then, the cationic lipid (2,3-Dioleoyloxy-propyl)-trimethylammonium-chloride (DOTAP) and helper lipid 1,2-dioleoyl-sn-glycero-3-phosphoethanolamine (DOPE) with different adding ratios were involved in the preparation of CLPs (Table S1). The obtained CLPs formulations P_1_-P_6_ were subjected to Zeta Potential and safety evaluation. As shown in Fig. 1A, the cationic surface was shown in P_4_-P_6_ but not in P_1_-P_3_, indicating that DOTAP content greater than 3 mg is essential to construct CLPs. Nevertheless, the greater the DOTAP contents, the worse the safety for normal cells in the respiratory system. Thus, the effect of CLPs on the normal human bronchial epithelial cells Base-2b viabilities was determined to evaluate their safety (Fig. 1B). Considering that the encapsulated ATRA in 100 µg/mL CLPs could meet the safety requirements (Fig. S5), P_5_ was chosen as the optimized CLPs formulation. Then, the proposed CLPs were further modified with RGD peptide and TAT peptide to construct RGD-TAT-CLPs (Fig. 1C). As shown in Fig. 1D and Figs. S6-S7, the modification of the peptide did not obviously change the particle size, polydispersity index (PDI) and surface Zeta Potential. No significant change of ATRA EE was observed as well, demonstrating the satisfied drug loading capacity of RGD-TAT-CLPs (Fig. 1E). As shown in the transmission electron microscope (TEM) images, an evident shell structure was shown in the peptides-modified CLPs, in which RGD-TAT-CLPs showed the thickest shell, probably due to the dual peptide modifications (Fig. 1F). Then, the storage stability of RGD-TAT-CLPs was also studied based on the particle size, PDI and Zeta Potential measurements, which reflected the perfect stability within 15 days (Fig. 1G and Figs. S8-S9).

Having successfully established the RGD-TAT-CLPs, ATRA and miR-34a were loaded to construct the RGD-TAT-CLPs/ATRA@miR-34a. The ATRA encapsulation ratio was optimized as 1:20 according to the highest EE of about 88.37% and desired drug release profile (Table S2 and Fig. S10). Then, the miR-34a was adsorbed on the optimized formulation with different RGD-TAT-CLPs/ATRA:miR-34a ratios. As shown in Fig. 1H, the unloaded free miR-34a was measured by a gel electrophoresis analysis. Only when the ratio was higher than 10:1 could the miR-34a be completely adsorbed, and the formulation of 20:1 could also exhibit the desired particle size and positive Zeta Potential (Fig. 1I-1J). Thus, the RGD-TAT-CLPs/ATRA:miR-34a ratio of 20:1 was selected as the optimized formulation. Compared to free miR-34a, the stability of the encapsulated miR-34a in fetal bovine serum (FBS) was extraordinarily improved (Fig. 1K). After incubation of 12 h, the miR-34a could still maintain stability while the free miR-34a was completely degraded within 1 h. Taken together, the RGD-TAT-CLPs/ATRA@miR-34a was successfully prepared, which co-loaded ATRA and miR-34a and improved the stability of miR-34a.

### Cellular uptake and in vitro antitumor effect

**Fig. 2:**
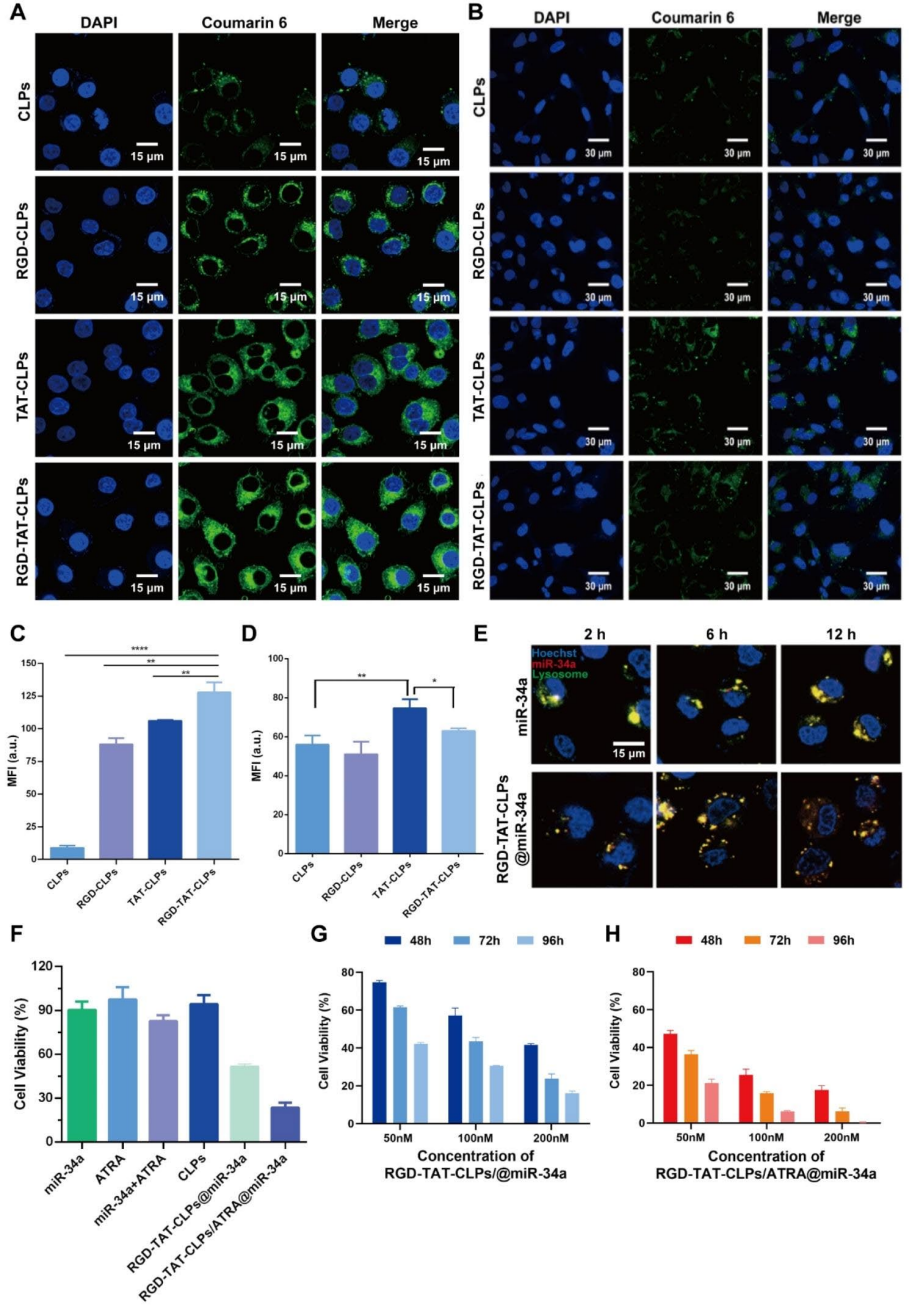
Cellular uptake and in vitro antitumor effect of RGD-TAT-CLPs/ATRA@miR-34a: (**A-B**) Cellular uptake of different formulations in A549 (**A**) and Beas-2b cells (**B**), where green fluorescence represents different CLPs while blue fluorescence represents cell nucleus. (**C-D**) Semi-quantitative analysis of A (**C**) and B (**D**). (**E** The CLSM co-localization images of RGD-TAT-CLPs@miR-34a and miR-34a with A549 cells lysosomes at different time points. (**F**) Cell viability of A549 cells with different treatments. (**G-H**) In vitro A549 cell growth inhibition effect of RGD-TAT-CLPs@miR-34a (**G**) and RGD-TAT-CLPs/ATRA@miR-34a (**H**) with different miR-34a concentrations under different concentrations and time points. (*n* = 6)

After successful preparation of the RGD-TAT-CLPs/ATRA@miR-34a, the cellular uptake and in vitro antitumor effects were evaluated using human lung tumor cells A549. Equipped with the dual-peptide modifications, the proposed nanosystem was anticipated to efficiently target tumor cells and be internalized. To demonstrate delivery specificity, normal human bronchial epithelial cells (Beas-2b) were employed for comparative analysis. The liposomes were fluorescently labeled with coumarin 6 to facilitate tracking of their cellular distribution. As shown in Fig. 2A and 2C, in A549 cells, the intracellular green fluorescence in peptide-modified CLPs groups was much greater than CLPs groups, proving the higher CLPs uptake amounts induced by RGD and TAT modifications. As anticipated, the RGD-TAT-CLPs group demonstrated the most robust green fluorescence, exhibiting approximately 14.34 times higher intensity compared to the CLPs group. This observation highlights the synergistic effects of RGD’s tumor cell targeting capacity and TAT’s membrane permeation ability. However, it is noteworthy that in Beas-2b cells, while the RGD modification did not significantly enhance cellular uptake efficiency, a slight improvement was observed with the TAT modification in terms of CLPs’ cellular uptake level (Fig. 2B and 2D). These results indicated that the RGD could specifically identify the tumor cells while the TAT could enhance cellular internalization even in non-tumor cells. With this superior tumor cell targeting and uptake capacity, more ATRA and miR-34a were expected to be intracellularly delivered, boosting the antitumor gene therapy.

However, as another intracellular delivery challenge of nanosystems, the lysosome capture and sequestration might severely compromise their therapeutical efficiency [7]. Thus, the lysosome escape ability of the proposed RGD-TAT-CLPs@miR-34a was evaluated. As shown in Fig. 2E, the colocalization of miR-34a and lysosomes were imaged. Compared to free miR-34a, the co-localization of RGD-TAT-CLPs@miR-34a and lysosome sequentially decreased as time went by, indicating the lysosome escape ability of RGD-TAT-CLPs. It could be explained by the proton-sponge effect of cationic DOTAP in CLPs. After being captured by lysosomes, the DOTAP could be protonated and induce the chloride ions influx, which might lead to the osmotic swelling and the physical rupture of the lysosomal membrane.

Subsequently, the in vitro antitumor effect of the proposed RGD-TAT-CLPs/ATRA@miR-34a was determined. As shown in Fig. 2F, the free miR-34a could not induce an obvious antitumor effect, which may be attributed to their instability in the cell culture medium. The free ATRA could neither exhibit a cell-killing effect due to its low working concentration. When the A549 cells were treated with the combination of miR-34a and ATRA, a stronger cytotoxicity was shown, indicating that ATRA could enhance the antitumor effect of miR-34a by augmenting the GJ functions to boost the intercellular transport of miR-34a. Stunningly, when miR-34a was encapsulated in the RGD-TAT-CLPs, the cell cytotoxicity was significantly improved with cell viability decreasing from about 90.36% to about 51.53%. This could be ascribed to the enhanced stability and cellular uptake of RGD-TAT-CLPs@miR-34a. Expectably, the strongest cell-killing effect was observed in the RGD-TAT-CLPs/ATRA@miR-34a group, suggesting the gene therapy promotion effect of the GJ regulating strategy.

Then, the concentration-dependent and time-dependent in vitro tumor growth inhibition effect of RGD-TAT-CLPs@miR-34a (Fig. 2G) and RGD-TAT-CLPs/ATRA@miR-34a (Fig. 2H) was further investigated. As time went on, the cell viability in all the groups obviously decreased, indicating the time-dependent manner. Compared to that of RGD-TAT-CLPs@miR-34a, the cell growth inhibition effect of RGD-TAT-CLPs/ATRA@miR-34a group was much greater with about only 0.35% cell viability in 96 h under 200 nM, demonstrating the desired antitumor effect of RGD-TAT-CLPs/ATRA@miR-34 assisted by the GJ regulating strategy. These results collaboratively proved the enhanced antitumor effect by dual peptide-modified CLPs plus GJ function regulation.

### Gene therapy enhancement mechanism by GJ regulating strategy

**Fig. 3:**
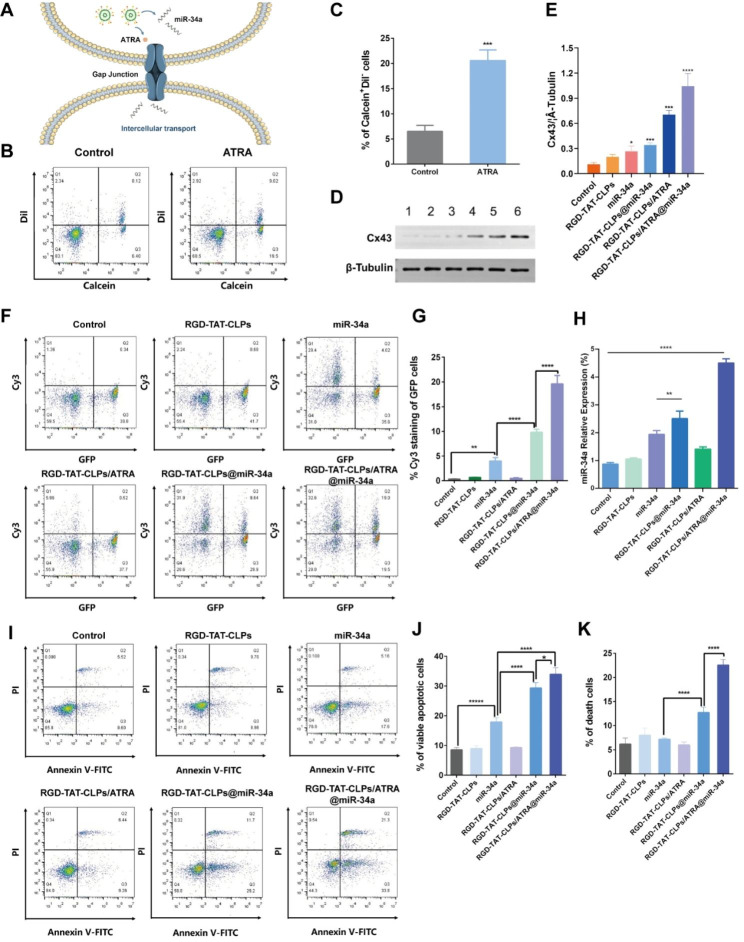
Gene therapy enhancement mechanism by GJ regulation strategy: (**A**) Schematical illustration of the mechanism of GJ regulation enhanced miR-34a transport. (**B-C**) The flow cytometry results of donor and recipient A549 cells labeled with Calcein-AM and Dil-CM staining (**B**) and its semi-quantitative analysis of Calcein^+^Dil^−^ cells (**C**). (**D-E**) The Western blot results of Cx43 in A549 cell after different treatments (**D**) and its semi-quantitative analysis (**E**), (1) Control, (2) RGD-TAT-CLPs, (3) miR-34a, (4) RGD-TAT-CLPs@miR-34a, (5) RGD-TAT-CLPs/ATRA, (6) RGD-TAT-CLPs/ATRA@miR-34a. (**F-G**) The intercellular transport of miR-34a in A549 cells analyzed with flow cytometry (**F**) and its semi-quantitative analysis of GFP^+^Cy3^+^ cells (**G**). (**H**) The miR-34a expression level in A549 cells with different treatments. (**I-K**) The Annexin V-FITC/PI flow cytometry results of A549 cells treated with different treatments (**I**) and its semi-quantitative results (**J** and **K**) (*n* = 3)

According to the abovementioned results, the proposed RGD-TAT-CLPs/ATRA@miR-34a exhibited an extraordinary antitumor effect in the A549 cell model, which was attributed to the outcome of the GJ regulating strategy. When the RGD-TAT-CLPs/ATRA@miR-34a was intracellularly delivered, the GJ-relative Cx was expected to be upregulated by ATRA, which in turn enhanced the GJ-mediated miR-34a intercellular transport by the “by-stander effect”. The widespread miR-34a in tumor cells could induce cell apoptosis (Fig. 3A). To consolidate this hypothesis, a series of experiments were carried out.

Firstly, the GJ existence and the function thereof were evaluated by a flow cytometry method, where the “donor cells” stained with Calcein-AM and Dil-CM were seeded into the blank “recipient cells”. Calcein-AM could only be transported between donor and recipient cells through GJ while Dil-CM could not be intercellularly transported. Thus, the percentage of the Calcein^+^Dil^−^ cells could be employed to analyze the GJ functions. As shown in Fig. 3B and 3C, the ATRA treatment significantly increased the Calcein^+^Dil^−^ cells from about 6.4% to about 19.5%, indicating the enhancement of GJ in A549 cells. Then, the expression of Cx43, a critical GJ constituting Cx, was investigated with different treatments to further validate the GJ-regulating effect of RGD-TAT-CLPs/ATRA@miR-34a (Fig. 3D and 3E). Surprisingly, the miR-34a and RGD-TAT-CLPs@miR-34a treatments slightly upregulate the expression of Cx43, which might be ascribed to the complicated signaling mechanism. Besides, the Cx43 expression in RGD-TAT-CLPs/ATRA and RGD-TAT-CLPs/ATRA@miR-34a group was much higher than other groups, indicating the payload could upregulate the Cx43 expression to enhance the GJ function. Subsequently, the effect of the enhanced GJ function on miR-34a intercellular transport was determined by treating the “recipient cells” A549 transfected with a green fluorescent protein (GFP-A549) plus “donor cells” A549 cells treated with different Cy3-labelled miR-34a formulations (Fig. 3F and 3G). Thus, the intercellular miR-34a transport could be revealed by the percentage of Cy3^+^GFP^+^ cells. As shown, the free miR-34a treatment could slightly increase the Cy3^+^GFP^+^ cells percentage from about 0.34% to about 3.98%, indicating the weak GJ functions of A549 cells and the instability of free miR-34a. However, the encapsulation of miR-34a into RGD-TAT-CLPs remarkably upregulated the miR-34a level in GFP-A549 cells, which might be the result of miR-34a stability enhancement. Expectedly, the highest Cy3^+^GFP^+^ cells percentage was exhibited in the RGD-TAT-CLPs/ARTA@miR-34a group, demonstrating the superior effect of GJ regulating strategy on miR-34a intercellular transport. These findings collectively support the notion that miR-34a can be transported between cells through GJ, a process further enhanced by ATRA co-delivery.

Then, the relative miR-34a expression of the abovementioned A549-GFP recipient cells was investigated. As shown in Fig. 3H, similar to the results of miR-34a intercellular transport analysis, the improved miR-34a expression levels were observed in RGD-TAT-CLPs@miR-34a and RGD-TAT-CLPs/ATRA@miR-34a groups, and the highest expression level was shown in the latter. These results further validated the feasibility of dual-peptide modified CLPs and the GJ regulating strategy in the enhanced delivery of miR-34a. Afterward, the effects of miR-34a expression on cell apoptosis were studied. It was reported that the p53-related cell apoptosis signaling pathway might be triggered by the miR-34a expression. The Annexin V-FITC/PI flow cytometry method was employed to analyze the percentage of viable apoptotic cells (Annexin V-FITC^+^PI^−^ cells) and death cells (Annexin V-FITC^+^PI^+^ cells). As shown in Fig. 3I-K, miR-34a treatment enhanced the viable apoptotic cell percentage from 8.6 to 17.9%, indicating its apoptosis-inducing function. Nevertheless, much significant cell apoptosis (about 29.2%) and death (about 11.7%) were induced by the RGD-TAT-CLPs@miR-34a, demonstrating the enhanced stability and delivery of miR-34a. The most effective cell apoptosis and cell death were caused by the RGD-TAT-CLPs/ATRA@miR-34a group, suggesting the boosted cell-killing efficiency induced by GJ regulation mediated miR-34a expression. Taken together, the miR-34a-induced gene therapy was validated to be boosted by the ATRA causing GJ regulation and dual-peptide modified CLPs delivery.

Furthermore, in accordance with the gap junction regulatory strategy, it is noteworthy that a strategically designed sequential release system could potentially exhibit a profound antitumor effect, wherein the administration of ARTA would precede that of miR-34a. In this scenario, the initial release of ARTA would effectively upregulate Cx43 expression to facilitate gap junction opening, subsequently enabling the subsequent release of miR-34a to traverse through tumor cells and induce apoptosis. This innovative design will be subject to forthcoming investigation.

### In vivo anti-tumor effect and mechanism on transplant subcutaneous Tumor model

**Fig. 4:**
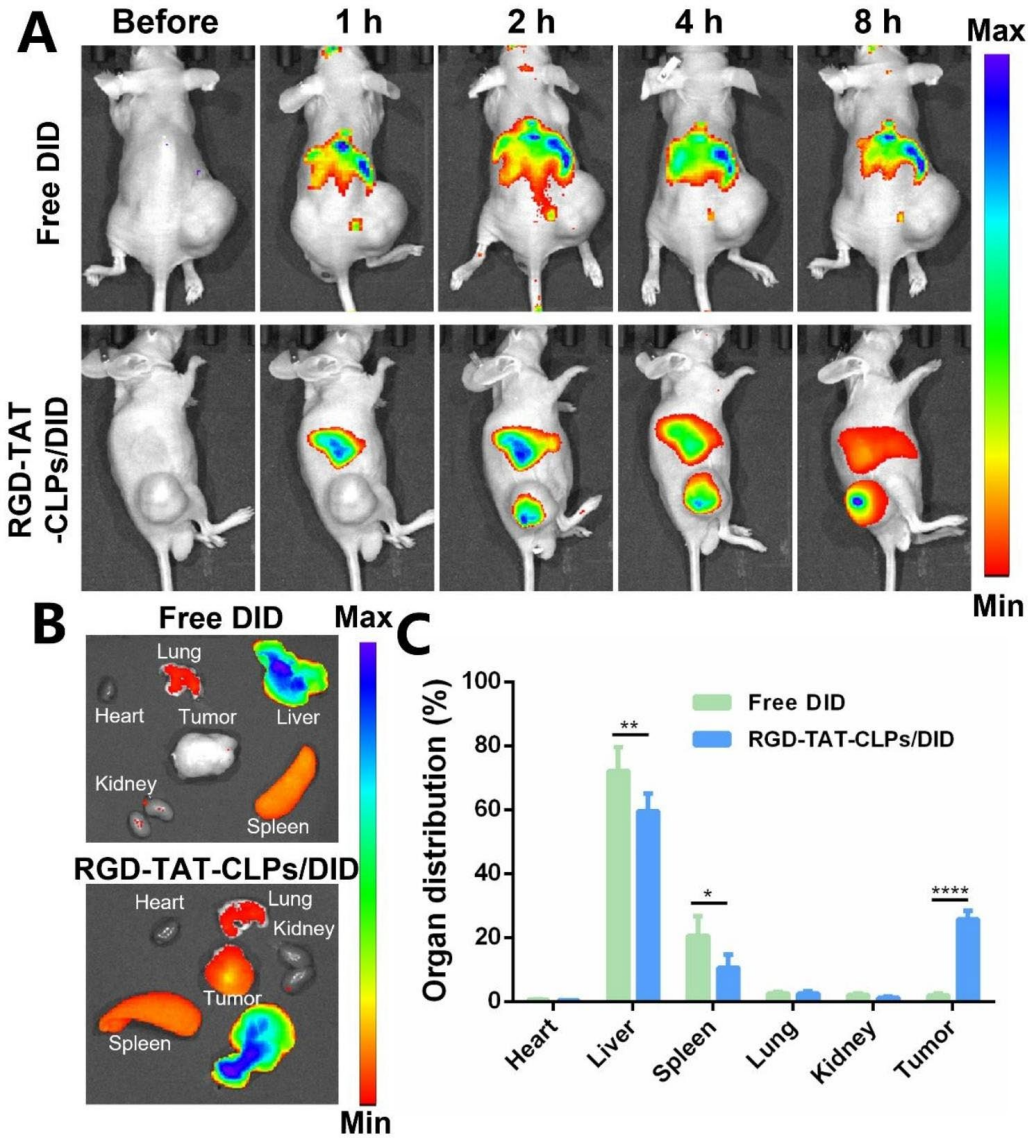
In vivo targeting ability evaluation of RGD-TAT-CLPs: (**A**) The fluorescence images of subcutaneous tumor-bearing mice after the intravenous injection of free DID or RGD-TAT-CLPs/DID at different times. (**B-C**) The fluorescence image (**B**) and its semi-quantitative analysis results (**C**) of major organs and tumors derived from tumor-bearing mice at 8 h. (*n* = 3)

The desired in vitro anti-tumor effect of RGD-TAT-CLPs/ATRA@miR-34a inspired us to evaluate its in vivo antitumor effect, which was first validated on a transplant subcutaneous A549 tumor model. Before evaluating the tumor growth inhibition efficiency, the in vivo tumor-targeting ability of dual-peptide modifications was investigated. The fluorescence probe DID was labeled on the RGD-TAT-CLPs to trace their in vivo accumulation, and the free DID group was set as a comparative group (Fig. 4). After intravenous injection, the RGD-TAT-CLPs/DID quickly accumulated in the tumor site within 2 h while the tumor fluorescence in the free DID group was not obvious (Fig. 4A). The high tumor accumulation in RGD-TAT-CLPs/DID group was maintained until 8 h, indicating its superior tumor targeting capacity. A similar conclusion could be drawn from the ex vivo results, where the highest DID accumulation was recorded in tumors except for the liver in RGD-TAT-CLPs/DID groups while no distinct tumor targeting ability was found in the free DID group (Fig. 4B and 4C). In addition, the CLPs/DID group was further added to evaluate the function of dual-peptide modification (Fig. S11). A significantly enhanced tumor drug accumulation was also exhibited in the RGD-TAT-CLPs/DID group compared to the CLPs/DID group. These results could be explained by the tumor cell targeting capacity of RGD and the cellular transmembrane capacity of TAT. Taken together, RGD-TAT-CLPs could serve as an effective carrier to deliver drugs to tumor lesions, facilitating the antitumor effect.

**Fig. 5:**
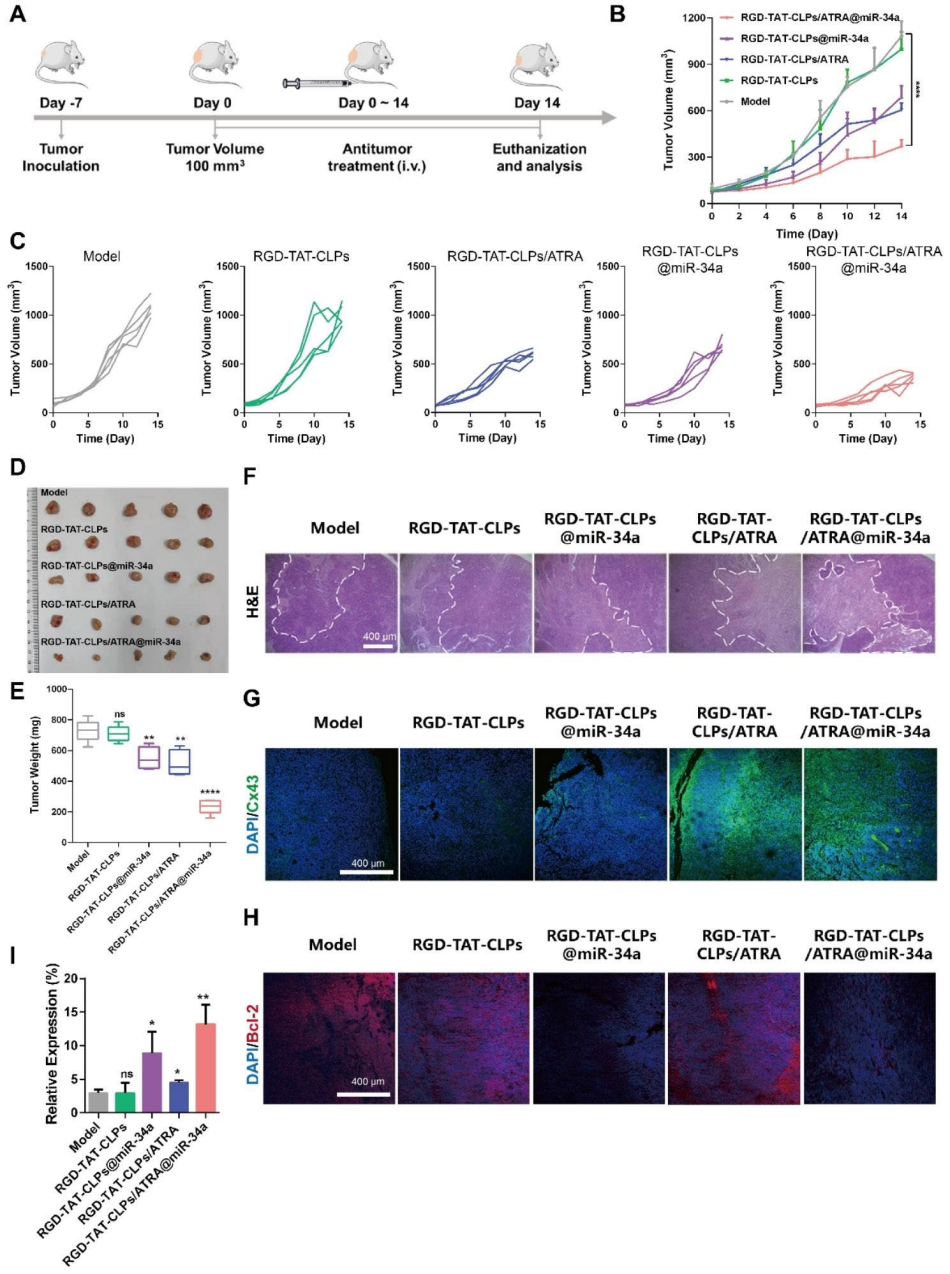
In vivo anti-tumor effect of RGD-TAT-CLPs/ARTA@miR-34a: (**A**) Schematical illustration of in vivo antitumor effect evaluation in transplant subcutaneous A549 tumor model. (**B-C**) A549 tumor volume curves of mice in 14 days with different treatments. (**D-E**) Tumors images (**D**) and average weights (**E**) of tumors dissected from tumor-bearing mice on the 14th day after different treatments. (**F-H**) H&E staining (**F**), Cx43 staining (**G**) and Bcl-2 (**H**) staining images of tumors from mice with different treatments. (**I**) The relative expression of miR-34a in tumors after different treatments on the 14th day. (*n* = 5)

Then, the in vivo antitumor effects were evaluated (Fig. 5A). There were five groups: Model, RGD-TAT-CLPs, RGD-TAT-CLPs/ARTA, RGD-TAT-CLPs@miR-34a and RGD-TAT-CLPs/ATRA@miR-34a. The tumor volume curves are shown in Fig. 5B and 5C. As shown, the tumor in the model group was enlarged in an exponential manner with the tumor volume exceeding 1000 mm^3^ on the 14th day. No obvious inhibition effect was exhibited in the RGD-TAT-CLPs group, indicating the negligible antitumor effect of the nanocarrier. However, tumor growth was significantly inhibited in the other three groups. The loading of ATRA or miR-34a into RGD-TAT-CLPs could to some extent exert some antitumor effect. The tumor inhibition effect of ATRA might be ascribed to its tumor plasticity-regulating ability while that of miR-34a was the result of its tumor apoptosis-inducing ability. Limited by the poor tumor cells penetration of miR-34a, the tumor inhibition effect of RGD-TAT-CLPs@miR-34a was not satisfied. In comparison, the strongest tumor-killing effect was shown in the RGD-TAT-CLPs/ARTA@miR-34a group, with the slowest increase in tumor volume in all mice and an average tumor volume of about 370 mm^3^ after 14 days, which might be attributed to the enhanced miR-34a delivery into deep tumors by the GJ regulating strategy. These results were confirmed by the tumor inhibition rate results (Fig. S12). To further validate the conclusion, the tumors were excised on the 14th day, which were then imaged and weighed. As shown in Fig. 5D and 5E, the lowest average tumor weight (about 235.00 mg) was recorded in the RGD-TATPCLPs/ARTA@miR-34a group, which was much lower than that in the model group (about 729.40 mg), suggesting a 2.78-time higher therapeutical efficiency than RGD-TAT-CLPs@miR-34a group. From the abovementioned results, the effectiveness of the GJ regulating strategy on gene therapy enhancement in vivo was strongly supported.

Afterward, the excised tumors were sliced for pathological examination to analyze their antitumor mechanism. According to the results of H&E staining, the highest tumor damaged area and the lowest tumor cells density were shown in the RGD-TAT-CLPs/ARTA@miR-34a group, indicating its strongest tumor cell-killing effect (Fig. 5F). To validate the delivery of GJ regulating strategy mediated miR-34a to deep tumor tissues, the Cx43 staining was employed to detect the GJ function in tumor tissues (Fig. 5G). As expected, the model group with low Cx43 expression exhibited impaired GJ function. However, treatment with RGD-TAT-CLPs/ARTA and RGD-TAT-CLPs/ARTA@miR34a significantly enhanced green fluorescence intensity, indicating upregulated Cx43 expression in tumor tissues. This observation further supports the notion of improved GJ functions. The augmented GJ function is likely to facilitate intercellular transport of miR-34a and subsequently induce tumor apoptosis. As revealed in the staining images of Bcl-2, a miR-34a-related apoptosis-inhibition protein, the apoptosis rate was remarkably enhanced in the RGD-TAT-CLPs@miR-34a and RGD-TAT-CLPs/ATRA@miR-34a groups, indicating the apoptosis-inducing effect of miR-34a (Fig. 5H). Finally, the miR-34a expression level in tumor tissues was quantitatively measured by qRT-PCR (Fig. 5I). Consistent with the in vitro results, the highest miR-34a expression level was revealed in the RGD-TAT-CLPs/ARTA@miR-34a group, which was about 4.49 times higher than the model group. The miR-34a expression was also higher than the RGD-TAT-CLPs@miR-34a group, suggesting the robustness of the GJ regulating strategy. These results collaboratively proved the great antitumor effects of the proposed RGD-TAT-CLPs/ARTA@miR-34a.

The safety of the proposed RGD-TAT-CLPs/ARTA@miR-34a was also evaluated during the therapeutical period. No obvious body weight change was recorded (Fig. S13). According to the organ weight efficiency analysis, the main organs including heart, liver, spleen, lung and kidney were not obviously damaged, which could also be found in the appearance (Figs. S14 and S15). Then, the main organs were excised for H&E analysis. As shown in Fig. S16, no noticeable abnormalities, such as fibrosis, infiltration, or inflammation, were found in the major organs of treated mice, indicating the good safety of the nanosystem. Finally, revealed by the hemogram examination, no significant impact of the proposed RGD-TAT-CLPs/ARTA@miR-34a on blood cells and functions was found (Fig. S17). Taken together, the presented RGD-TAT-CLPs/ARTA@miR-34a did not cause any observable systematical toxicity.

### Construction and characterization of RGD-TAT-CLPs/ARTA@miR-34a-DPIs

Given its desirable lung accumulation ability and low enzyme activity in the respiratory system, pulmonary delivery of gene therapeutic agents holds promise for exerting a more potent antitumor effect compared to other delivery routes for lung tumor treatment. In light of their solid-state form that enhances drug loading and maintains gene stability, DPIs have been recognized as an ideal pulmonary delivery system. Therefore, we aimed to develop DPI formulations of the synthesized RGD-TAT-CLPs/ARTA@miR-34a complex. For this purpose, lactose (Lac), an FDA-approved DPI carrier material, along with hydroxypropyl-β-cyclodextrin (HP-β-CD), a generally recognized as safe (GRAS) material by FDA, were selected as carrier materials for RGD-TAT-CLPs/ARTA@miR-34a-DPIs.

**Fig. 6:**
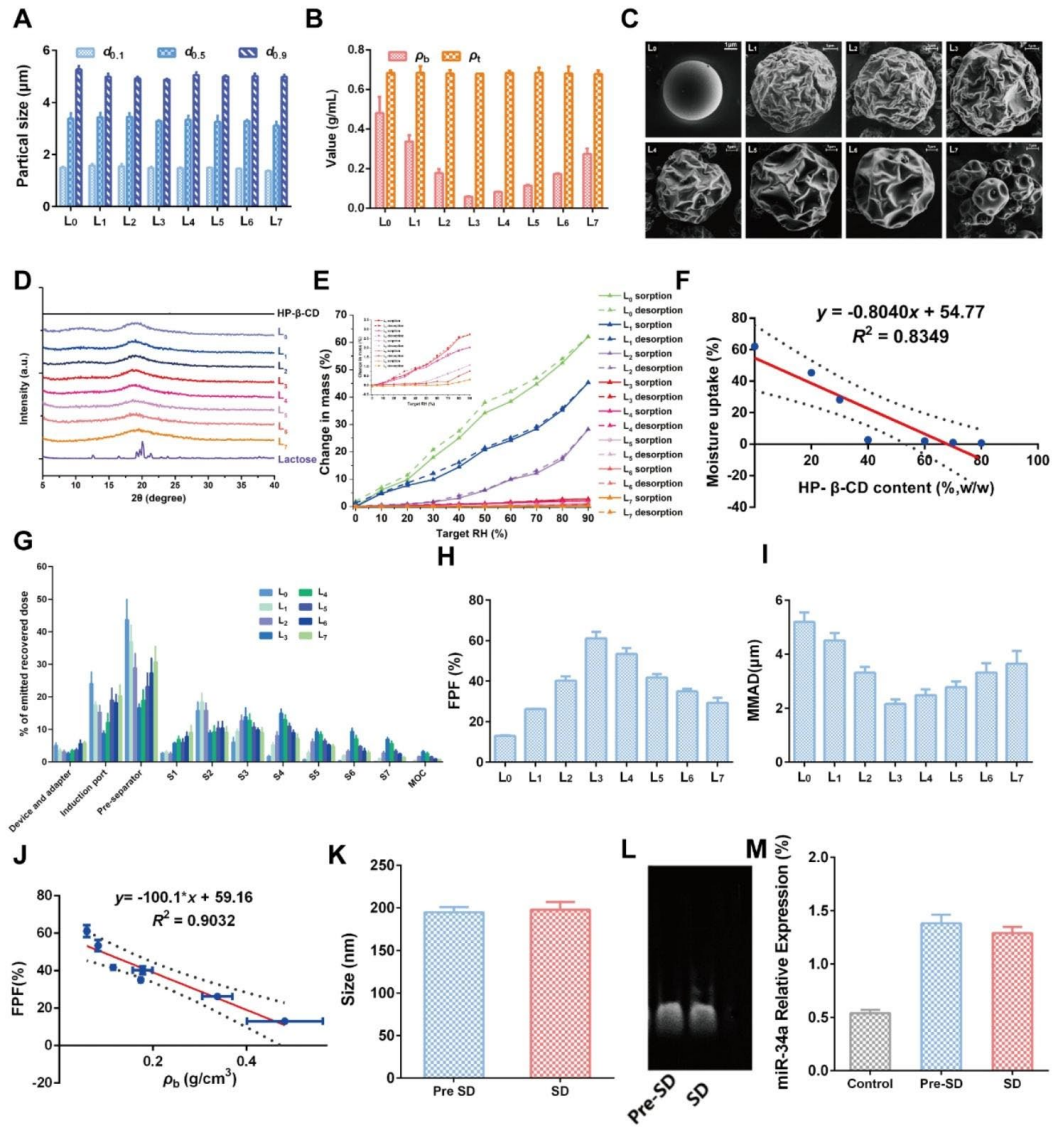
Construction and characterization of RGD-TAT-CLPs/ARTA@miR-34a-DPIs: (**A-E**) Particle size measurements (**A**), bulk density and tap density (**B**), surface morphology images (**C**), PXRD patterns (**D**) and water adsorption curves (**E**) of RGD-TAT-CLPs/ARTA@miR-34a-DPIs of different formulations. (**F**) The correlation between moisture uptake and the HP-β-CD content in RGD-TAT-CLPs/ARTA@miR-34a-DPIs. (**G**) The in vitro deposition distribution of RGD-TAT-CLPs/ARTA@miR-34a-DPIs of different formulations. (**H-I**) The FPF (**H**) and MMAD (**I**) of different formulations. (**J**) The correlation between FPF and *ρ*_b_ value of DPIs. **(K)** The particle size of RGD-TAT-CLPs/ARTA@miR-34a before and after SD. **(L-M)** The gel electrophoresis images (**L**) and qRT-PCR detection results (**M**) of RGD-TAT-CLPs/ATRA@miR-34a before and after SD

To obtain desired DPIs with satisfied aerosolization properties, a series of Lac- HP-β-CD ratios was set to prepare formulations L_0_-L_7_ by a spray drying (SD) method (Table 1). Then, the basic physicochemical properties of the prepared DPIs were investigated to determine the optimized formulation. As shown in Fig. 6A, the particle size of all the formulations was similar, with a *d*_0.1_ of about 1.5 μm and a *d*_0.9_ of about 5 μm, suggesting the great potential for delivery to the lower airway. It was considered that the appropriate particle size for inhalation should be between 0.5 and 5 μm, which would guarantee particles deposition in the deep lung after multiple deposition mechanisms like collision with the airway surface, gravitational sedimentation and diffusional deposition [39]. Then, the bulk density (*ρ*_b_) and tap density (*ρ*_t_) were measured (Fig. 6B). No obvious difference in *ρ*_t_ was observed while *ρ*_b_ decreased from L_0_ to L_3_ but increased from L_4_ to L_7_, indicating the addition of HP-β-CD might exert significant effects. Among them, the smallest *ρ*_b_ was recorded in L_3_, implying the best dispersibility and flowability. Further, the morphology of DPIs particles was observed by scanning an electronic microscope (SEM), and the results are shown in Fig. 6C. A perfect spherical and smooth particle was observed in L_0_ without HP-β-CD addition. With the addition of HP-β-CD, the particle surface became rougher and corrugated in L_1_-L_6_, which was more similar to the date stone-like morphology of the HP-β-CD carrier (L_7_). However, the surface morphology of L_4_-L_7_ was too corrugated to maintain the spherical shape, which might induce the particles to cross-link to each other, resulting in worse pulmonary delivery performance. Then, the crystallinity of DPIs was analyzed by Powder X-Ray Diffraction (PXRD). As shown in Fig. 6D, a typical α-crystallinity was found in Lac while HP-β-CD exhibited an amorphous state. For DPIs, no obvious diffraction peaks were found in all the formulations, indicating that the DPIs particles were in the amorphous state. Besides, the hygroscopicity of DPIs was determined as water adsorption might significantly impact the DPIs particles stability, in turn influencing their aerosolization performance. The weight gain of L_0_ was about 50.7% at 90% RH, suggesting the great hygroscopicity of Lac carrier (Fig. 6E). Nevertheless, with the addition of HP-β-CD, the water adsorption was remarkably inhibited with less than 5% in L_1_-L_7_, which might be attributed to the moisture-resistance nature of HP-β-CD. It was revealed that the hygroscopicity was negatively correlated with the HP-β-CD addition amount (Fig. 6F). The above results collaboratively demonstrated that the proper addition of the HP-β-CD could enhance the potential pulmonary delivery performance of DPIs by increasing the surface roughness and inhibiting water adsorption, while L_1_-L_3_ may possess the best performance.

Then, the in vitro aerosolization performance of RGD-TAT-CLPs/ARTA@miR-34a-DPIs was investigated by the next generation impactor (NGI), and the results are shown in Fig. 6G. The small geometric standard deviation (GSD) values of all formulations suggested a narrow particle size distribution (Fig. S18). Compared to other formulations, a much higher lower airway deposition (S3 - S7) and a decreased deposition in the device and adaptor, induction port, pre-separator and S1 - S2 were revealed in L_3_. Further, the highest (about 61.17%) fine particle fraction (FPF) and appropriate (about 2.16 μm) mass median aerodynamic diameter (MMAD) was also validated in L_3_. Herein, FPF was defined as the fraction of drug effectively deposited in the lung, while MMAD was regarded as the aerodynamic size of particles with a mass cumulative percentage of 50% (Fig. 6H and I) [40]. These results strongly supported that the optimized pulmonary delivery performance would be achieved by L_3_. And a negative correlation between the FPF value and the *ρ*_b_ of DPIs was also revealed (*R*^2^ = 0.9032), which was consistent with other reports (Fig. 6J) [40].

Having constructed the optimized RGD-TAT-CLPs/ARTA@miR-34a-DPIs, the drug loading capacity and stability were investigated before and after the SD process. The size and surface Zeta Potential of loaded RGD-TAT-CLPs/ARTA@miR-34a were determined before and after SD (Fig. 6K and Fig. S19). It was revealed that no obvious change in size and Zeta Potential was observed before and after SD, indicating the great recoverability of RGD-TAT-CLPs/ARTA@miR-34a when delivered into the lung. Then, the stability of the encapsulated drugs was analyzed before and after the SD process. As shown in Fig. 6L, the activity of loaded miR-34a was not impacted by the SD process with similar electrophoresis images. Further, the qRT-PCR results revealed that the relative expression level of miR-34a was not influenced by the SD process, which was still much higher than the control group (Fig. 6M). In addition, the ATRA loading and its release profile were also maintained after the DPIs construction process (Fig. S20). Taken together, a negligible impact of the SD process on the stability and drug loading of RGD-TAT-CLPs/ARTA@miR-34a was found, indicating its high feasibility to serve as a pulmonary delivery platform for antitumor gene therapy.

Based on what we discussed above, the proposed RGD-TAT-CLPs-DPIs were equipped with great pulmonary delivery performance and desired gene delivery stability. Thus, it was believed that the presented DPIs system could serve as a pulmonary gene delivery platform. For further in vitro and in vivo studies, more gene therapy would be supported by the proposed DPIs platform for the treatment of other lung local diseases.

**Table 1:** Formulation of carrier solution with different ratios of Lac-HP-β-CD

Formulation	L_0_	L_1_	L_2_	L_3_	L_4_	L_5_	L_6_	L_7_
Lactose (%, w/w)	100	80	70	60	40	30	20	0
HP-β-CD (%, w/w)	0	20	30	40	60	70	80	100

## Conclusions

In conclusion, the proposed RGD-TAT-CLPs/ATRA@miR-34a was proven to overcome the three bottlenecks of lung cancer gene therapy, the poor tumor tissue penetration effect, the insufficient lung drug accumulation and unsatisfied gene transfection efficiency. The dual peptide modifications demonstrate superior tumor-targeting properties by enhancing tumor site recognition and cellular uptake. Furthermore, intercellular miR-34a transport is elevated through GJ function regulation mechanism to boost gene transfection efficiency in deep tumors. In vitro and in vivo studies have confirmed the remarkable anti-tumor effects of RGD-TAT-CLPs/ATRA@miR-34a, which has been engineered into DPIs using carrier materials Lac and HP-β-CD. The engineered DPIs were found to be equipped with a rough surface and desired moisture-resistance properties, which were expected to improve deep lung deposition and lung drug accumulation, making it a promising pulmonary gene delivery platform. This study provides valuable insights into the administration of gene therapy agents *via* the respiratory system, thus contributing significantly to the advancement of innovative treatments for lung tumors.

## Materials and methods

### Materials

The EP, Lyso-Tracker Green, DAPI and Hoechst 33,342 were purchased from Solarbio Life Science (Beijing, China). The Chol was provided by MYM Biological Technology (Beijing, China). DOTAP and DOPE was provided by Corden Pharma (Zurich, Switzerland). ATRA, Cx43 antibody and β-Tubulin antibody were supplied by Sigma-Aldrich (Shanghai, China). miR34a was purchased from Shanghai GenePharma Co.,Ltd (Shanghai, China). DSPE-PEG_5000_-RGD and DSPE-PEG_2000_-TAT was provided by Xi’an ruixi Biological Technology (Xi’an, China). Lipfectamine 2000® was purchased from Thermo Fisher Scientific (Waltham, MA). Cell counting kit-8 (CCK8) was obtained from Dojindo Laboratories (Kumamoto, Japan). Annexin V-FITC apoptosis detection kit was acquired from Beyotime Biotechnology (Shanghai, China).

### Preparation and characterization of RGD-TAT-CLPs

The RGD-TAT-CLPs was prepared by a thin layer dispersion method. Firstly, the blank liposomes were optimized and prepared according to different formulations and process parameters. The blank liposomes prepared by different EP/Chol ratios, hydration volumes, ultrasonic powers and ultrasonic times were subject to size and Zeta Potential measurements to optimize the formulation, respectively. Then, the EP/DOTAP/DOPE/CHOL ratios were further screened to construct blank CLPs. Then, the blank CLPs were modified by DSPE-PEG_5000_-RGD and DSPE-PEG_2000_-TAT to obtain RGD-TAT-CLPs.

The particle diameters and surface Zeta Potential of the prepared RGD-TAT-CLPs were measured using the DLS method with a Malvern Mastersizer 2000 (Malvern Instruments Ltd., UK). The morphology of blank CLPs, RGD-PEG_5000_-CLPs, TAT-PEG_2000_-CLPs and RGD-TAT-CLPs were further confirmed by TEM using an electron microscope (JEM-100 CX II, JEOL Ltd., Tokyo, Japan).

### Preparation and characterization of RGD-TAT-CLPs/ATRA@miR-34a

Having successfully constructed the RGD-TAT-CLPs, the ATRA and miR34a were further loaded to prepare RGD-TAT-CLPs/ATRA@miR-34a. The ATRA was added to the lipid phase while the miR34a was spontaneously adsorbed on the surface. The ATRA loading capacity was determined by an ultrafiltration centrifugation method (4000 rpm, 30 min). The collected free ATRA were determined by HPLC method. In addition, the miR34A loading capacity was measured by an agarose gel electrophoresis method.

The stability of free and encapsulated miR34a were evaluated after being incubated in the medium of 10% FBS under 37℃. The samples were processed by Triton X-100 to release free miR34a, which was subject to agarose gel electrophoresis to determine the remaining contents. The ATRA release profile was evaluated by a dialysis method. About 1 mL of the RGD-TAT-CLPs/ATRA@miR-34a was put into a dialysis bag (MWCO: 3500 Da), which was soaked in about 40 mL PBS buffer with 0.5% Tween 80. The system was incubated at 100 rpm under 37 °C. Then 2 mL of release medium was taken out at the indicated time intervals to quantify the released ATRA amount.

### Cellular uptake assay and lysosome Escape evaluation

The cellular uptake of RGD-TAT-CLPs/ATRA@miR-34a was studied by a confocal laser scanning microscopy (CLSM) method in two cell models, A549 and Beas-2b. Briefly, the loaded drug was replaced by C-6 to trace the liposomes. The cells were seeded in a glass-bottom 24-well plate at a density of 5 × 10^3^ cells per well for 24 h. Then, the culture medium was replaced by the fresh medium containing C6-CLPs, C6-TAT-CLPs, C6-RGD-CLPs and C6-RGD-TAT-CLPs for 4 h. Then, the cells were washed by PBS and fixed by 4% paraformaldehyde for 30 min. Then the redundant paraformaldehyde was washed by PBS, and the cell nuclei were stained with DAPI for 10 min. Finally, the cells were observed and imaged using a CLSM (FV3000, Olympus, Tokyo, Japan), and the data were further analyzed by ImageJ software.

The lysosome escape was studied by a similar method. The miR34a was labeled by Cy3, lysosomes were stained by Lyso-Tracker Green and nuclei was stained by Hoechst 33,342. The samples were subject to CLSM, and the co-localization was analyzed by ImageJ.

### In vivo targeting capacity

To investigate the In vivo targeting ability of RGD-TAT-CLPs/ATRA@miR-34a, the A549 tumor-bearing mouse model was constructed. Briefly, the BALB/C nu/nu (male, 18 − 22 g) was provided by the Guangdong Medical Laboratory Animal Center. All animal experiments were conducted following the guidelines of laboratory animals supervised by Institutional Animal Care and Use Committee of Sun Yat-Sen University (SYSU-IACUC-2022-000326). The A549 cell suspensions were mixed with Matrigel with the concentration about 6 × 10^7^ mL^− 1^, which was subcutaneously injected into the back of mice. The tumor volumes (*V*) were determined by an electronic caliper and calculated as (tumor length) × (tumor width)^2^/2.

When the tumor volume reached 400 mm^3^, the mice were intravenously injected with DID-loaded RGD-TAT-CLPs/ATRA@miR-34a. Then, the mice were imaged at 2, 4, 6 and 8 h via a small animal imaging system (IVIS Lumina Series III, PerkinElmer, USA). Then, the mice were sacrificed, and the main organs and tumor were excised and imaged. The data was further analyzed by ImageJ.

### In vitro antitumor efficiency assay

The In vitro antitumor efficiency was evaluated by cytotoxicity assay and Annexin V-FITC/PI staining assay. As for cytotoxicity assay, the A549 cells were seeded in a 96-well plate at a density of 5 × 10^3^ cells per well for 24 h. After treated with different formulations for 24 h, the cell viability of cells was determined by CCK8 assay.

For Annexin V-FITC/PI staining assay, a similar method was used. The A549 cells were seeded in a 6-well plate at a density of 5 × 10^5^ cells per well for 24 h. After treated with different formulations for 24 h, the cells were collected and stained by Annexin V-FITC/PI according to the instructions (Beyotime Biotechnology, Shanghai, China), which was further analyzed by flow cytometer.

### GJ functions evaluation

The GJ functions were evaluated by a flow cytometry method. Briefly, the A549 cells were seeded in a 6-well plate at a density of 1.5 × 10^5^ cells per well until 90% confluent. Then, cells were divided into two groups. Cells in one group were stained with Calcein-AM and Dil-CM probes for 30 min and collected, which is denoted as “donor cell”. Then, the stained cells were inoculated into cells in another group (“recipient cell”). After 12 h incubation, the cells were collected and subjected to flow cytometry.

### Protein expression level of Cx43

The expression of Cx43 was determined by Western blot assay. Briefly, the A549 cells were treated with miR34a, ATRA, RGD-TAT-CLPs, RGD-TAT-CLPs@miR-34a and RGD-TAT-CLPs/ ATRA@miR-34a for 24 h, respectively. The treated cells were collected and lysed to obtain protein samples. Cx43 antibody and β-Tubulin antibody was employed to testify the samples following the western blot process.

### Intercellular miR34a transport evaluation

The intercellular miR34a transport was detected by a flow cytometry method. A549 cells were seeded in a 6-well plate at a density of 1 × 10^5^ cells per well and cultured for 24 h. Then, the cells were treated with Cy3 labeled miR-34a, RGD-TAT-CLPs@miR-34a, RGD-TAT-CLPs/ATRA@miR-34a, RGD-TAT-CLPs/ATRA and RGD-TAT-CLPs, respectively. Among them, the Cy3 labeled miR34a group was transfected by a commercial vector Lipfectamine 2000®. After 12 h, the cells were collected and inoculated in GFP-A549 cells for another 6 h. Then, the cells were collected for flow cytometry analysis.

### mRNA expression level of miR34a

The mRNA expression level of miR34a in treated cells was determined by a qRT-PCR method. Briefly, cells were treated with miR34a, RGD-TAT-CLPs, RGD-TAT-CLPs/ATRA, RGD-TAT-CLPs@miR-34a and RGD-TAT-CLPs/ATRA@miR-34a for 6 h, respectively. Then, the cells were collected, and the RNA was extracted. Then, the corresponding miRNA cDNA was synthesized using the M5 miRNA qPCR Assay Kit and M5 miRNA cDNA Synthesis Kit according to the instructions. Finally, the synthesized cDNA, forward primer, reverse primer and 2× M5 miRNA qPCR were mixed and subjected to qRT-PCR assay.

### In vivo antitumor efficiency

The A549 tumor-bearing mouse models were established as abovementioned. When the tumor volume reached 100 mm^3^, the A549 tumor-bearing mice were randomly divided into five treatment groups (*n* = 6): Control, RGD-TAT-CLPs, RGD-TAT-CLPs@miR-34a, RGD-TAT-CLPs/ATRA, and RGD-TAT-CLPs/ATRA@miR-34a. Then, the mice were treated with different formulations via *i.v.* injection every 3 days, and the tumor volume was measured every 2 days. On 14th day, the mice were sacrificed, and the tumors were excised, photographed and weighted. Then, the tumors were fixed and embedded for pathological analysis. Part of the tumors were sliced and stained with H&E, Blc-2 and Cx43 to analyze the i*n vivo* antitumor mechanism. Meanwhile, the other part of the tumors was homogenized to extract their RNA, which was then subjected to qRT-PCR to analyze the miR-34a expression level.

### Biocompatibility assay

At the end of the treatment period, blood samples were collected by the method of enucleation to analyze the hemogram. The main organs including heart, liver, spleen, lung, and kidney of mice were excised and weighted. Then the organs were fixed in 4% paraformaldehyde. The organs were embedded and sliced for H&E staining. The slices were observed and imaged by an optical microscope.

### Preparation of RGD-TAT-CLPs/ATRA@miR-34a-DPIs

The prepared RGD-TAT-CLPs/ATRA@miR-34a were further developed as a DPIs by a spray drying method. The FDA-approved material Lac and FDA GRAS material HP-β-CD were chosen as DPIs carriers. The carrier materials were mixed with different ratios to obtain a carrier solution of 20 mg/mL, which was spray dried with RGD-TAT-CLPs/ATRA@miR-34a of 100 µg/mL using Eyela SD-1000 spray-dryer (Tokyo Rikakikai Co., Ltd., Tokyo, Japan) with a nozzle diameter of 0.71 mm. The spray-dryer was operated under the following conditions: atomization pressure 90 kPa, feed rate 10 mL/min, air flow 0.60 m^3^/h, inlet temperature 100 °C, and outlet temperature 65 °C.

### Characterization of RGD-TAT-CLPs/ATRA@miR-34a-DPIs

Then, the prepared DPIs with different lactose/HP-β-CD ratios were characterized and optimized according to particle size distribution, bulk density and tap density, surface morphology, crystallinity, hygroscopicity and aerosolization performance. The particle size distribution (*d*_0.1_, *d*_0.5_ and *d*_0.9_) was analyzed by Malvern laser diffraction particle size analyzer (Mastersizer 2000, Malvern Instruments Ltd., UK). Measurements were carried out using a dry dispersion system with a dispersion pressure of 3.5 bar. *Span* was calculated by Eq. 1.

1 $$Span = {\text{ }}\left( {{d_{0.9}} - {d_{0.1}}} \right)/{d_{0.5}}$$

The, the bulk density (*ρ*_b_) and tap density (*ρ*_t_) of RGD-TAT-CLPs/ATRA@miR-34a-DPIs were evaluated by a graduated cylinder according to a reported method [40]. Then, the DPIs were visualized and imaged by a Gemini 500 Scanning electron microscopy (SEM, Gemini 500, Bruker Co., Bremen, Germany) to evaluate its surface morphology. In addition, the Powder X-ray diffraction (PXRD, D8 Quest, Bruker Corporation, Bremen, Germany) was employed to analyze the crystallinity of DPIs.

Then, the hygroscopicity of DPIs was determined by dynamic vapor sorption (DVS, Aquadyne DVS, Quantachrome Corporation, USA). When determining the water adsorption curves, the relative humidity (RH) was set between 5% and 90% while the humidity gradient was set at 10% RH. The original mass was recorded as *m*_0_ and the sample mass when moisture absorption equilibrium reach 90% RH was recorded as *m*_1_. The weight gain percentage was calculated according to Eq. 2.

2 $${\text{Weight}}\,{\text{gain}}\,{\text{Percentage}}\, = \,\frac{{{m_1} - {m_0}}}{{{m_0}}} \times 100\%$$

### In vitro aerosolization performance of RGD-TAT-CLPs/ATRA@miR-34a-DPIs

The in vitro aerosolization performance of RGD-TAT-CLPs/ATRA@miR-34a-DPIs was evaluated by the Next generation impactor (NGI, Copley Scientific Ltd., Nottingham, UK). Briefly, the DPIs powders of about 10 mg were put into the 3# capsules. Then, the capsules were inserted into a Turbospin® device (PH&T S.p.A., Milan, Italy) which was connected to NGI. During measurement, the flow rate was set at 60 ± 2% L/min and the run time was set as 4 s. Then, the samples on the device and adaptor, induction port, pre-separator, and all NGI stages were carefully collected by anhydrous ethanol, which was then subjected to HPLC analysis to evaluate the drug concentrations. The critical parameters including fine particle fraction (FPF), mass median aerodynamic diameter (MMAD), and geometric standard deviation (GSD) was calculated by CITDAS® software (version 3.10, Copley Scientific Ltd., Nottingham, UK).

### Liposome recoverability of RGD-TAT-CLPs/ATRA@miR-34a-DPIs

To evaluate the liposome recoverability after spray drying, the particle size and Zeta Potential, appearance, Tyndall effect, drug loading, drug release and miR34a content were characterized. Briefly, the DPIs were dissolved in PBS and subjected to analysis as abovementioned, which was compared with the free RGD-TAT-CLPs/ATRA@miR-34a. When analyzing the miR34a contents, the DPIs were dissolved and the released liposomes were processed by Triton X-100, which were then subjected to agarose gel electrophoresis to determine the remaining contents.

### Statistical analysis

Statistical analysis was performed using GraphPad Prism 6.0. ANOVA or *t* tests were utilized to determine whether there were significant differences between the data, and a value of *p* < 0.05 was considered significant. All of the above experiments were measured in three times except for special instructions. *P* value style: *p < 0.05; **p < 0.01; ***p < 0.005; ****p < 0.001.

### Electronic supplementary material

Below is the link to the electronic supplementary material.

**Supplementary Material 1**: Additional file1. Additional figures and tables

## Data Availability

The data that support the findings of this study are available from the corresponding author upon reasonable request.
