# Supplementary material for: Dual peptides-modified cationic liposomes for enhanced Lung cancer gene therapy by a gap junction regulating strategy
Source: J Nanobiotechnology. 2023 Dec 9;21:473. doi: 10.1186/s12951-023-02242-1 (PMC10709977; doi:10.1186/s12951-023-02242-1)
Supplement: Supplementary file 1 — Supplementary Material 1: Additional file1. Additional figures and tables [file 12951_2023_2242_MOESM1_ESM.docx]

**Supporting information**

Table. S1 Formulation composition of CLPs

| Formulation | P_1_ | P_2_ | P_3_ | P_4_ | P_5_ | P_6_ |
| --- | --- | --- | --- | --- | --- | --- |
| EP (mg) | 14 | 13 | 12 | 10 | 8 | 6 |
| DOTAP (mg) | 1 | 1.5 | 2 | 3 | 4 | 5 |
| DOPE (mg) | 1 | 1.5 | 2 | 3 | 4 | 5 |
| CHOL (mg) | 4 | 4 | 4 | 4 | 4 | 4 |

Table. S2 The EE and DL of RGD-TAT-CLPs@miR-34a

with different ATRA loading ratio (*n* = 3)

| Formulation | EE (%) | DL (%) |
| --- | --- | --- |
| F1 (1:10) | 82.80 ± 4.73 | 2.85 ± 0.15 |
| F2 (1:20) | 88.37 ± 3.99 | 2.81 ± 0.07 |
| F3 (1:30) | 82.78 ± 1.21 | 1.79 ± 0.03 |


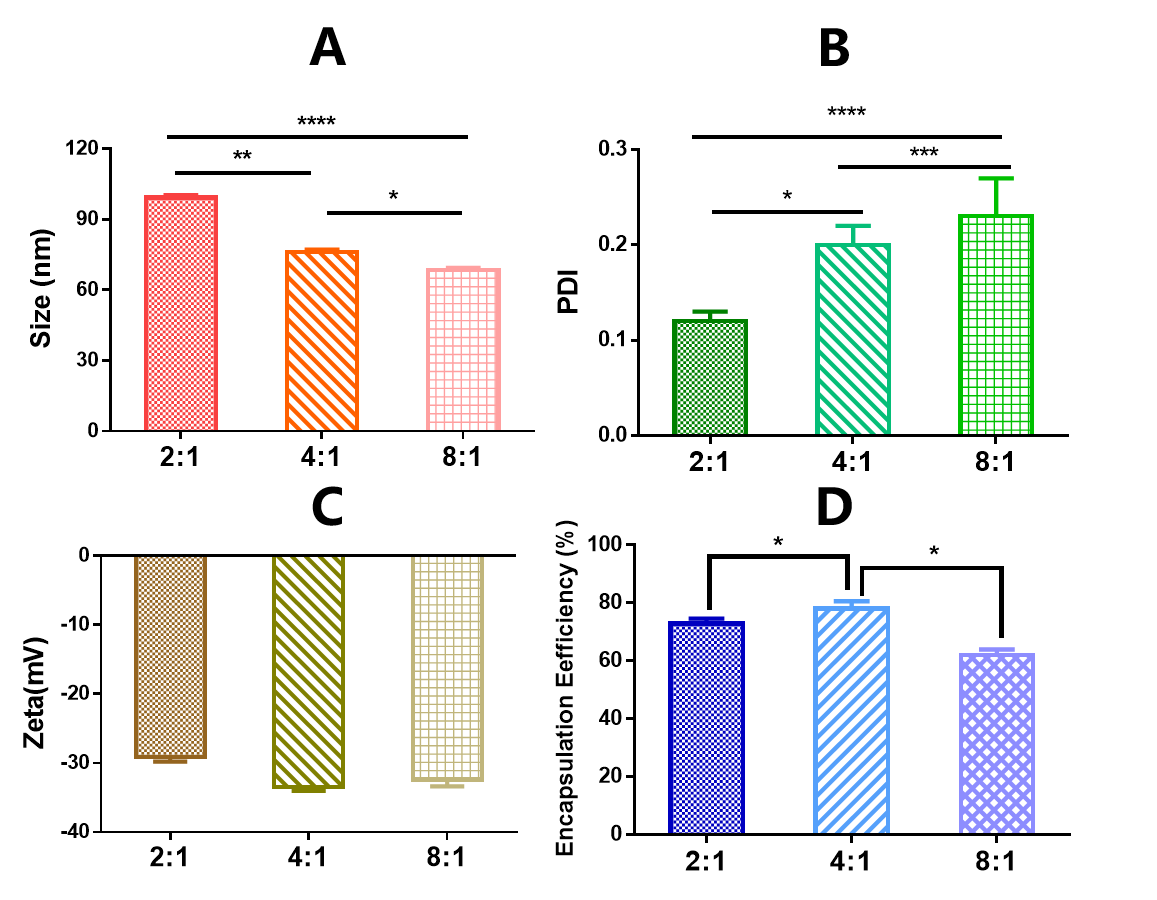


**Figure S1.** Effect of EP to CHOL ratio on the LPs size (A), PDI (B), Zeta potential (C) and EE (D) (*n* = 3)


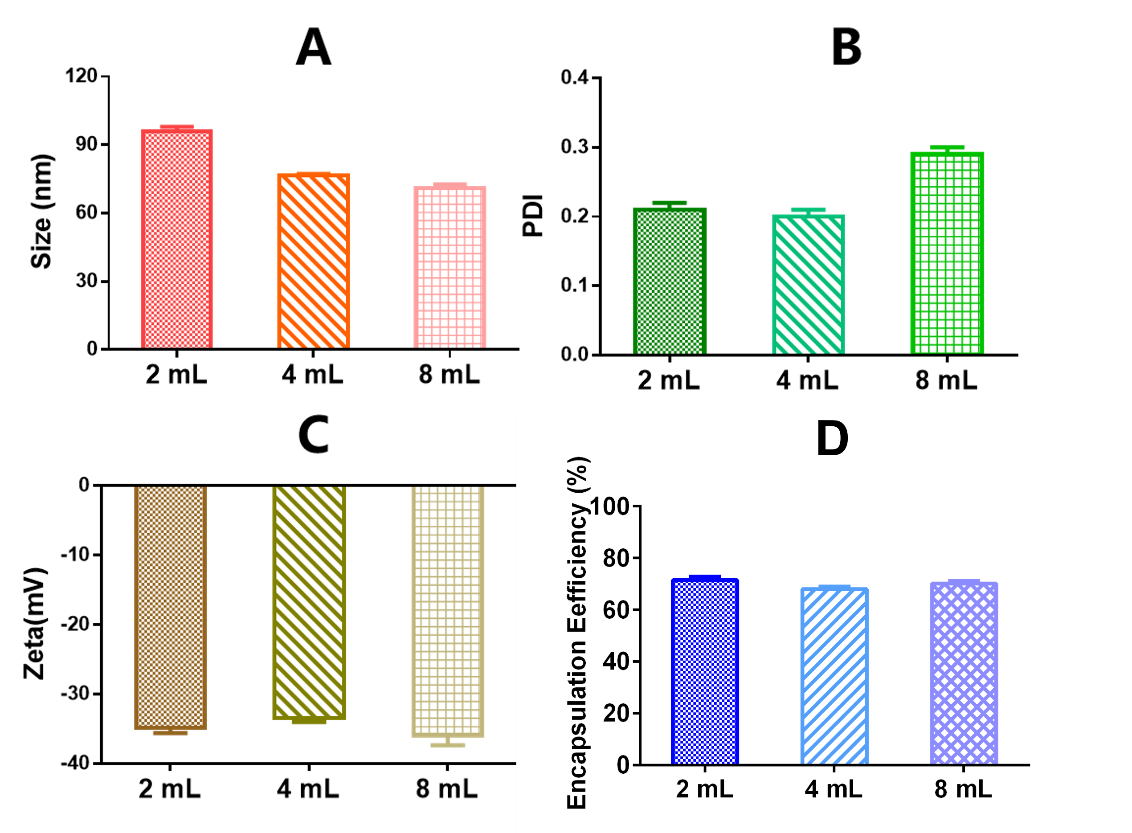


**Figure S2.** Effect of the hydration volume on the LPs size (A), PDI (B), Zeta potential (C) and EE (D) (*n* = 3)


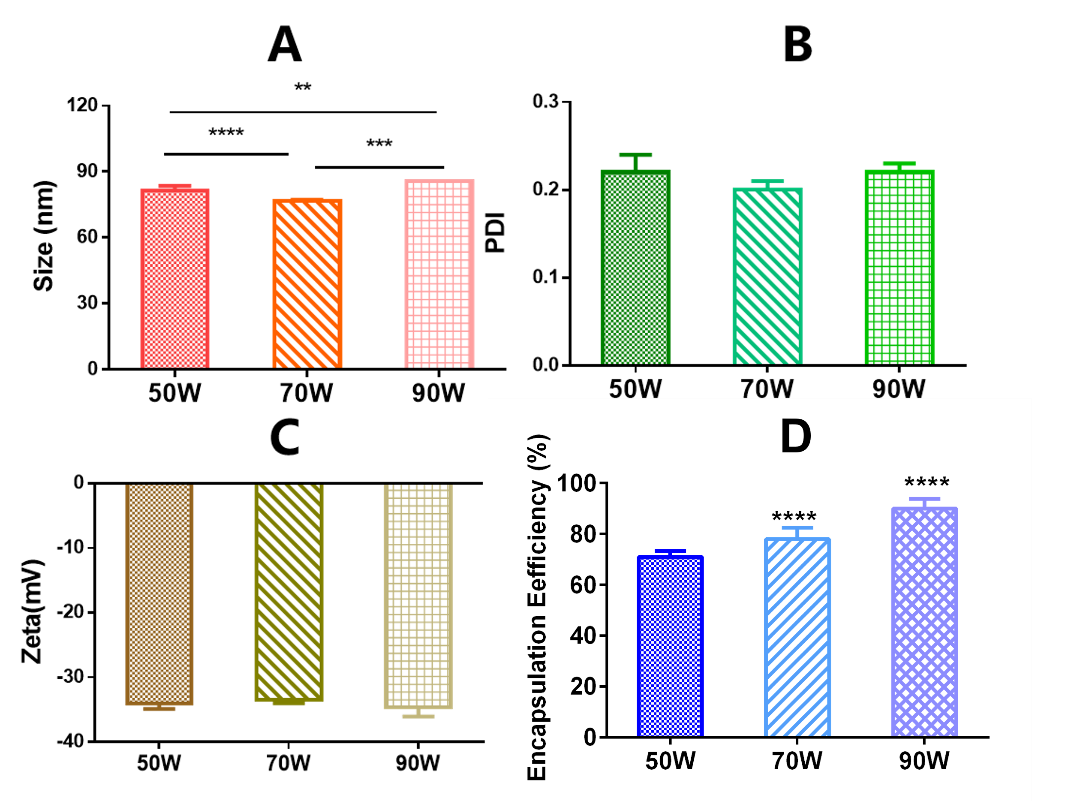


**Figure S3.** Effect of the ultrasonic power on the LPs size (A), PDI (B), Zeta potential (C) and EE (D) (*n* = 3)


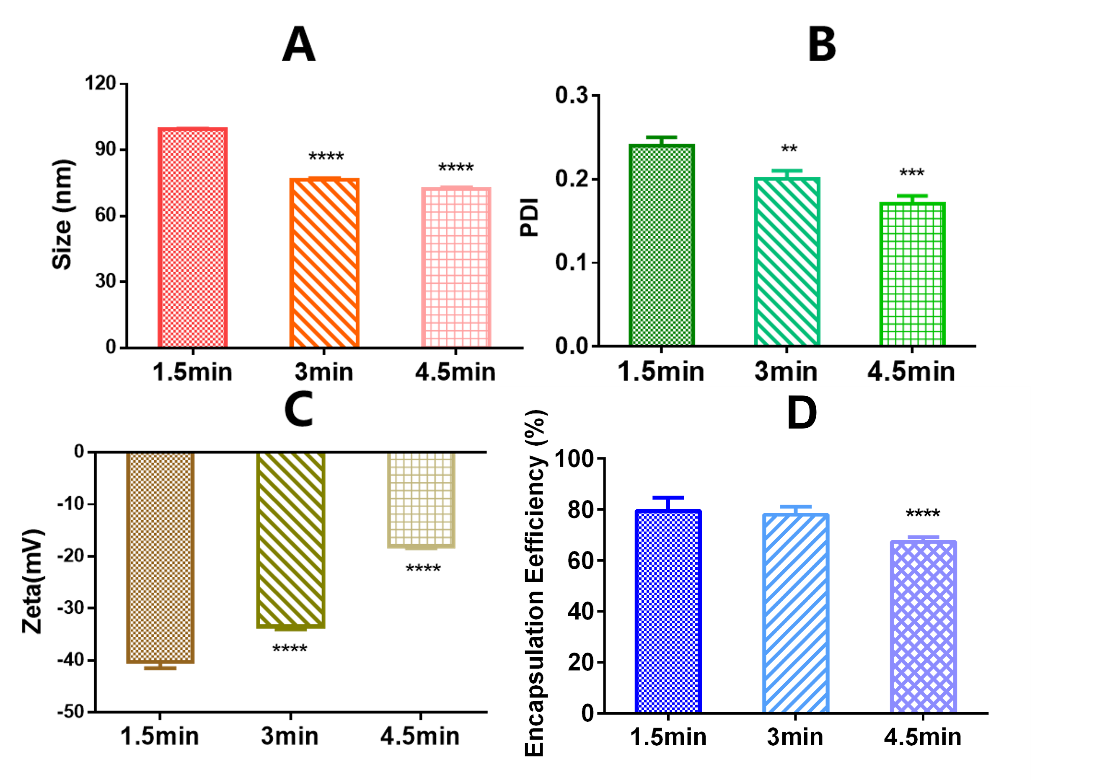


**Figure S4.** Effect of the ultrasonic time on the LPs size (A), PDI (B), Zeta potential (C) and EE (D) (*n* = 3)





**Figure S5.** Cytotoxicity test results of ATRA. (*n* = 6)





**Figure S6.** The PDI of CLPs, RGD-PEG_5000_-CLPs, TAT-PEG_2000_-CLPs and RGD-TAT-CLPs. (*n* = 3)





**Figure S7.** The Zeta potential of CLPs, RGD-PEG_5000_-CLPs, TAT-PEG_2000_-CLPs and RGD-TAT-CLPs. (*n* = 3)





**Figure S8.** The PDI stability of RGD-TAT-CLPs. (*n* = 3)





**Figure S9.** The zeta potential stability of RGD-TAT-CLPs. (*n* = 3)





**Figure S10.** Drug cumulative release profile of RGD-TAT-CLPs/ATRA@miR-34a with different ATRA loading ratio (*n* = 3)


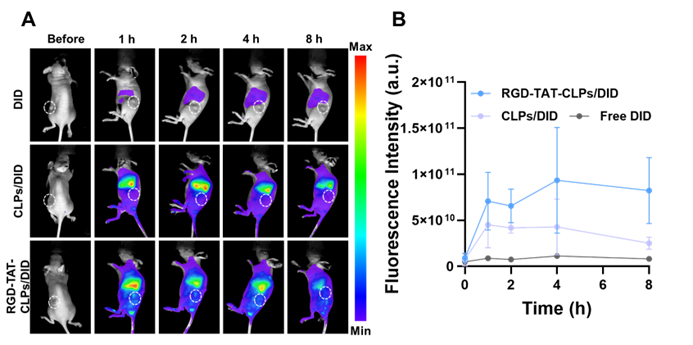


**Figure S11.** (A) The fluorescence images of subcutaneous tumor-bearing mice after the intravenous injection of free DID, CLPs/DID or RGD-TAT-CLPs/DID at different times. (B) The semiquantitative analysis of the fluorescence images. (*n* = 3)





**Figure S12.** The tumor inhibition rate (B) of A549 subcutaneous tumor after treatment with formulations on BALB/c Nude mice (*n* = 6)





**Figure S13.** The average weight of BALB/c Nude mice after different treatments on the 14th day (*n* = 6)


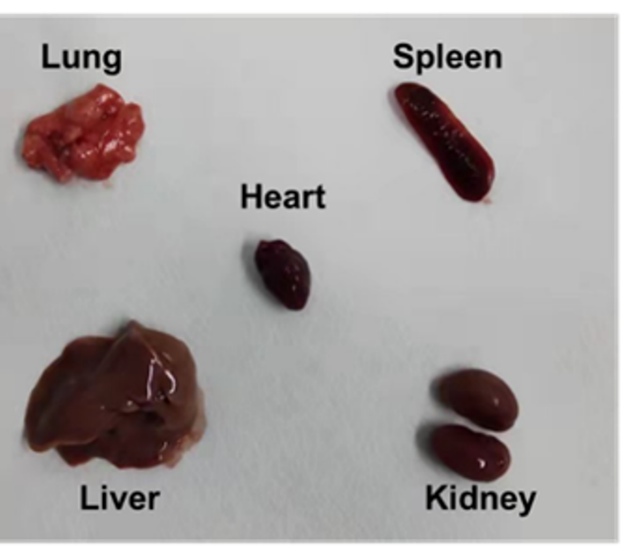


**Figure S14.** Major organs picture of BALB/c Nude mice after different treatments on the 14th day (*n* = 6)





**Figure S15.** Major organs viscera index of BALB/c Nude mice after different treatments on the 14th day (*n* = 6)


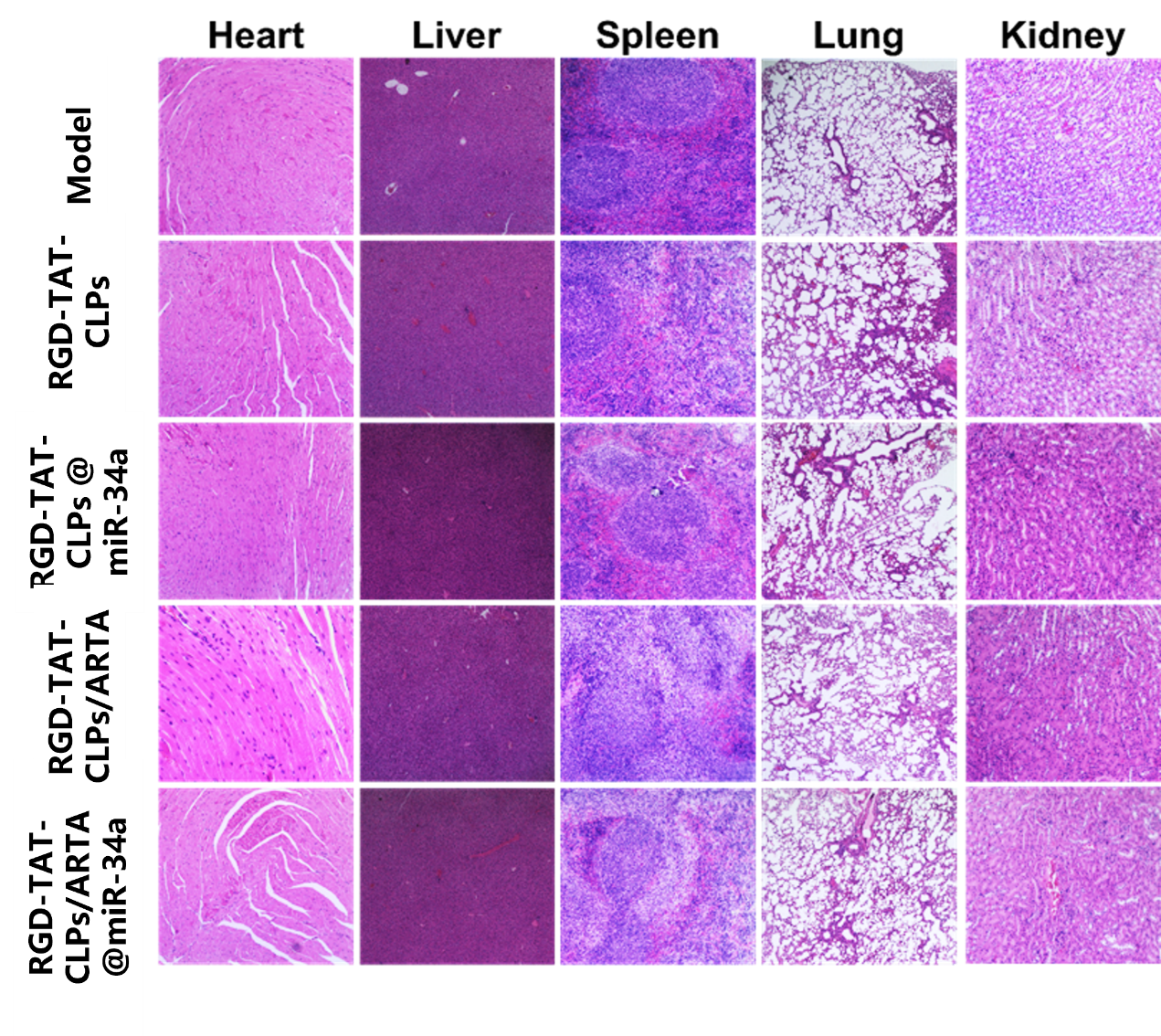


**Figure S16.** Histological images of BALB/c Nude mice after different treatments on the 14th day


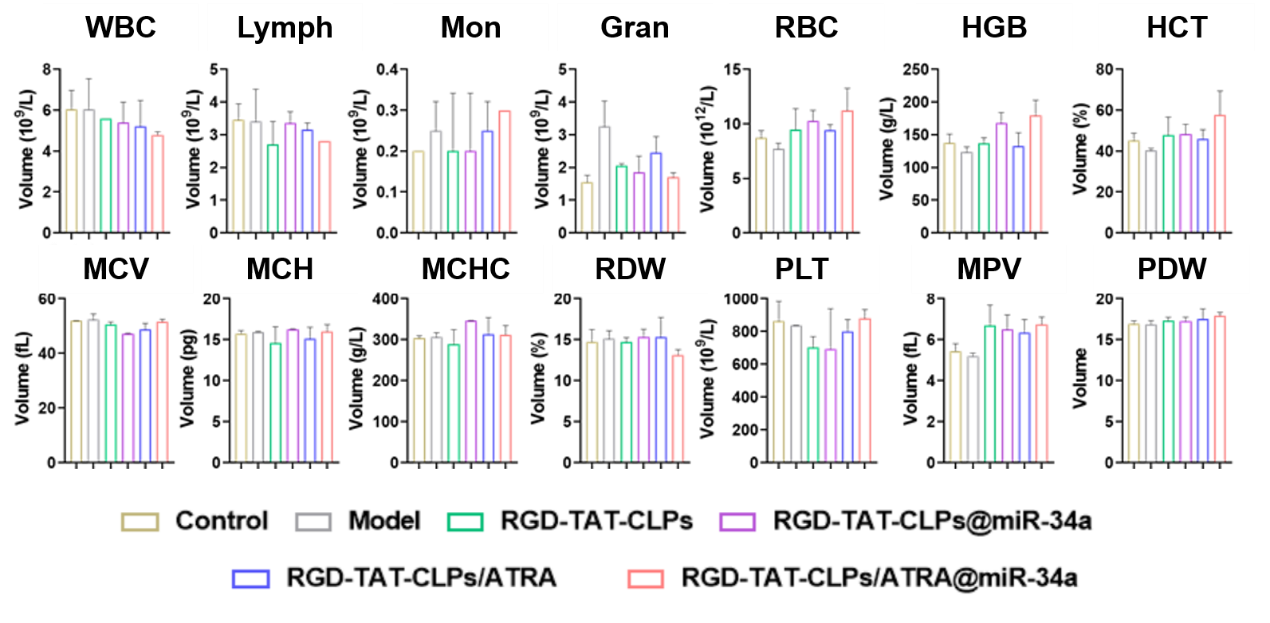


**Figure S17.** The blood routine test results of BALB/c Nude mice after different treatments on the 14th day (*n* = 6)





**Figure S18.** The GSD of different DPIs formulations. (*n* = 3)





**Figure S19.** The zeta potential of RGD-TAT-CLPs/ARTA@miR-34a before and after SD. (*n* = 3)





**Figure S20.** The drug loading and release profile of ATRA in RGD-TAT-CLPs/ATRA@miR-34a before and after spray drying (*n* = 3)
